# Genetic characterization of *Neisseria meningitidis* isolates recovered from patients with invasive meningococcal disease in Lithuania

**DOI:** 10.3389/fcimb.2024.1432197

**Published:** 2024-10-14

**Authors:** Anželika Slavinska, Magdalena Kowalczyk, Agnė Kirkliauskienė, Greta Vizuje, Paweł Siedlecki, Joana Bikulčienė, Kristina Tamošiūnienė, Aurelija Petrutienė, Nomeda Kuisiene

**Affiliations:** ^1^ Department of Microbiology and Biotechnology, Institute of Biosciences of Vilnius University Life Sciences Centre, Vilnius, Lithuania; ^2^ Institute of Biochemistry and Biophysics, Polish Academy of Sciences, Warsaw, Poland; ^3^ Faculty of Medicine, Institute of Biomedical Science, Vilnius University, Vilnius, Lithuania; ^4^ Microbiology Laboratory, Republican Vilnius University Hospital, Vilnius, Lithuania; ^5^ Saisei Medical Center, Saisei LT, Vilnius, Lithuania; ^6^ Department of Clinical Investigations of the National Public Health Surveillance Laboratory, Vilnius, Lithuania

**Keywords:** *N. meningitidis*, antimicrobial resistance, virulence, pathogenicity, whole-genome sequencing, genotyping

## Abstract

**Introduction:**

*Neisseria meningitidis* is a gram-negative bacterium responsible for life-threatening invasive infections known as invasive meningococcal disease and is associated with high fatality rates and serious lifelong disabilities among survivors.

**Methods:**

This study aimed to characterize *N. meningitidis* isolates cultured from blood and cerebrospinal fluid collected between 2009 and 2021 in Lithuania, assess their genomic relationships with European strains, and evaluate the possibility of using a cost-effective method for strain characterization, thus improving the national molecular surveillance of invasive meningococcal disease. In total, 321 *N. meningitidis* isolates were collected and analyzed using multilocus restriction typing (MLRT). Amplification of the *penA* gene and restriction fragment length polymorphism analysis were performed to identify the modified *penA* genes. Based on the MLRT genotyping results, we selected 10 strains for additional analysis using whole-genome sequencing. The sequenced genomes were incorporated into a dataset of publicly available *N. meningitidis* genomes to evaluate genomic diversity and establish phylogenetic relationships within the Lithuanian and European circulating strains.

**Results:**

We identified 83 different strains using MLRT genotyping. Genomic diversity of *N. meningitidis* genomes analysed revealed 21 different sequence types (STs) circulating in Lithuania. Among these, ST34 was the most prevalent. Notably, three isolates displayed unique combinations of seven housekeeping genes and were identified as novel STs: ST16969, ST16901, and ST16959. The analyzed strains were found to possess virulence factors not commonly found in *N. meningitidis*. Six distinct *penA* profiles were identified, each with different frequencies. In the present study, we also identified *N. meningitidis* strains with new *penA*, NEIS0123, NEIS1320, NEIS1525, NEIS1600, and NEIS1753 loci variants. In our study, using the cgMLST scheme, Minimum Spanning Tree (MST) analysis did not identify significant geographic relationships between Lithuanian *N. meningitidis* isolates and strains from Europe.

**Discussion:**

Discussion: To our knowledge, this is the first study to employ whole genome sequencing (WGS) method for a comprehensive genetic characterization of invasive *N. meningitidis* isolates from Lithuania. This approach provides a more detailed and precise analysis of genomic relationships and diversity compared to prior studies relying on traditional molecular typing methods and antigen analysis.

## 1 Introduction

*Neisseria meningitidis*, a prevalent gram-negative bacterial commensal in the human upper respiratory tract, is also capable of causing large epidemics of invasive meningococcal disease (IMD) (Stephens et al., 2007). *N. meningitidis*, which asymptomatically colonizes the nasopharynx in approximately 10% of healthy individuals, remains the main cause of meningitis and fulminant sepsis worldwide (Tzeng and Stephens, 2021). The transition from asymptomatic to invasive disease remains unclear. However, certain factors, such as the genetic and capsular structures of pathogenic strains, are thought to play a significant role (Mullally et al., 2021).

The distribution of serogroups of *N. meningitidis* shows significant variation worldwide, with six capsular types (A, B, C, W, X, and Y) responsible for most meningococcal disease cases (Lu et al., 2019). Although the capsule is the main factor determining the virulence of *N. meningitidis*, comparative genomic studies have revealed the presence of additional genomic factors in various lineages (Caugant and Maiden, 2009). Natural competence for genetic transformation and homologous recombination contribute to the extensive genomic diversity of meningococcal lineages, with recombination being the principal mechanism for introducing new genetic information (Schoen et al., 2009). Numerous factors have been linked to the pathogenesis of *N. meningitidis*, including increased invasiveness, overcoming host defenses, and resistance to antimicrobial treatment. Therefore, a comprehensive understanding of the adaptive and pathogenic mechanisms of *N. meningitidis* is important (Lu et al., 2019).

Molecular profiling of *N. meningitidis* is vital for global control of the disease. Genomic surveillance of bacterial meningitis pathogens facilitates the detection of emerging and spreading strains, leading to public health interventions (Rodgers et al., 2020). Over the past 10 years, in many European countries, epidemiological surveillance of *N. meningitidis* has been based on whole-genome sequencing (WGS) methods. The accessibility of WGS provides an opportunity to understand the biology and diversity of *N. meningitidis* (Bratcher et al., 2014). However, while WGS is employed in countries with the necessary capacity and resources, in middle- or low-income countries, not only is the WGS methodology not in use, but simpler genotyping techniques, such as multilocus sequence typing (MLST), are not applied. Typically, methods with low discriminatory power, such as slide agglutination serogrouping or polymerase chain reaction (PCR)-based techniques for serogroup prediction, are used for epidemiological surveillance of infectious diseases in Lithuania. Importantly, at the national level for routine surveillance of meningococcal disease, comprehensive typing methods like MLST, considered a principal method for detailed molecular characterization, are not routinely employed. MLST analysis is typically conducted retrospectively by research groups (Sereikaitė et al., 2023; Ivaškevičienė et al., 2023) rather than as part of standard surveillance protocols. This does not ensure proper identification and typing of pathogens isolated during outbreaks/epidemics, transmission dynamics, risk factor identification, pathogenesis, and the etiologic attribution of pathogens; thus, it is not known whether isolates causing invasive diseases belong to the same virulent strain or if they are different strains (Eybpoosh et al., 2017).

The epidemiology of IMD varies geographically and over time (Tzeng and Stephens, 2021) and is influenced by meningococcal vaccines and isolation measures employed during the COVID-19 pandemic in 2020–2021 (Alderson et al., 2022).

Compared to other European countries, the epidemiological situation in Lithuania has been challenging for several years. The number of IMD cases reported in Lithuania from 2009 to 2020 (median, 1.65 per 100,000 inhabitants) was higher than that reported in other European countries (median, 0.63 per 100,000 inhabitants). During the period from 2009 to 2012, the average notification rate of IMD cases was 1.47 cases per 100,000 population. From 2013 to 2017, the average rate increased to 2.20 cases per 100,000 population. Although 2018 showed a significant decrease in the number of notified IMD cases (1.10 cases per 100,000 population in 2017), it was marked by the highest case fatality rate in the past 10 years, reaching as high as 21.74%. A relatively high case fatality rate persisted in 2019 at 18.75%. The lowest number of notified IMD cases (0.25–0.39 per 100,000 population) and no associated fatalities were observed in 2020 and 2021 (European Centre for Disease Prevention and Control, 2023). Therefore, it is critically important to investigate the genotypic relationship of Lithuanian isolates with strains isolated in Europe to gain insights that can inform effective public health interventions.

## 2 Methods

### 2.1 Source of isolates

In total, 321 strains of *N. meningitidis* acquired from the National Public Health Surveillance Laboratory in Lithuania were examined. The isolates were collected from patients diagnosed with IMD between October 12, 2009, and August 25, 2021. Among them, 207 (64.5%) were isolated from blood and 114 (35.5%) from cerebrospinal fluid. Pure *N. meningitidis* cultures were stored at -80°C in tryptone soy broth containing 20% horse blood serum and 15% glycerol. To recover the isolates from long-term storage, 10 μL of freezing medium was transferred to Mueller–Hinton chocolate agar plates, and the isolates were incubated on the plates at 37°C with humidity for 18–24 h in an atmosphere of 5% CO2. A single colony from a visually pure culture of each *N. meningitidis* isolate was selected for subsequent analysis.

### 2.2 Antibiotic susceptibility testing

Minimum inhibitory concentrations (MIC) for penicillin G, cefotaxime, rifampicin, and ciprofloksacin were determined using Liofilchem^®^ MIC gradient strips (Liofilchem S.r.l., Italy) and interpreted according to European Committee on Antimicrobial Susceptibility Testing (EUCAST) breakpoints v13.1 (The European Committee on Antimicrobial Susceptibility Testing, 2023). Antimicrobial susceptibility testing was performed on Mueller–Hinton agar supplemented with 5% defibrinated sheep blood (Liofilchem S.r.l., Italy) and incubated at 35–37°C in 5% CO2. For quality control, *Streptococcus pneumoniae* ATCC 49619 and *Escherichia coli* ATCC 25922 were used.

To compare the MIC values between the different groups, the Kruskal–Wallis test was applied. The data obtained were analyzed using IBM SPSS statistics software (version 21), and statistical significance was set at p < 0.05.

### 2.3 Genomic DNA extraction

The total DNA of *N. meningitidis* strains was extracted from suspended single bacterial colonies using a GeneJET Genomic DNA Purification Kit (Thermo Fisher Scientific Inc.), following the manufacturer’s protocol. DNA quality was assessed by measuring absorbance at 260/280 nm, and integrity was verified using 1% agarose gel electrophoresis.

### 2.4 Characterization of isolates

To confirm the identity of *N. meningitidis* and determine its serogroup, all isolates underwent PCR analysis. Meningococcus-specific genes (*porA* and *ctrA*) and genes specific to the serogroup (*sacD*, *siaD*, *cap29EH*, *wnmB*, *capZC*, *lcbB*, *xcbA*, *wnmA*, *synG*, and *galE/lipA*) were targeted as previously described (Zhu et al., 2012.). Amplification products were separated on a 1% agarose gel. Genogroups for all publicly available *N. meningitidis* genomes from Lithuania were extracted from the pubMLST database.

All *N. meningitidis* strains were analyzed using multilocus restriction typing (MLRT), which was performed as described by Benett et al (Bennett and Cafferkey, 2003). MLRT involves the restriction fragment length polymorphism analysis of PCR products obtained from seven housekeeping gene loci used in MLST (Maiden et al., 1998). PCR products were digested with *Msp*I and *Mnl*I (Thermo Fisher Scientific Inc.) restriction endonucleases according to the manufacturer’s instructions and analyzed by 1% agarose gel electrophoresis. Alleles at each of the seven loci were combined to obtain an allelic profile or restriction type (RT). All RT were manually scored and analyzed. A dendrogram was constructed using Hierarchical Clustering—Unweighted Pair-Group Method with Arithmetic Averages (UPGMA) (Garcia-Vallvé et al., 1999) and visualized using interactive Tree of Life (iTOL) (Letunic and Bork, 2021).

Amplification of the *penA* gene and restriction fragment length polymorphism (RFLP) analysis were performed to identify the modified *penA* genes, following the method described by Antignac et al (Antignac et al., 2001). The *penA* PCR products were treated with restriction endonuclease *Taq*I (Thermo Fisher Scientific Inc.) according to the manufacturer’s instructions and assessed by 1% agarose gel electrophoresis. To evaluate the relationship between *penA* RFLP and *penA* sequencing methods, we analyzed data of publicly available isolates from Lithuania in the PubMLST database (Jolley et al., 2018). Additionally, we used a BLASTn search against the NCBI nr database to detect homology between rarely detected *penA* alleles and *penA* genes of other *Neisseria* species. To identify *penA* sequence differences between strains and mutations associated with penicillin resistance, we conducted a multiple sequence alignment using Jalview (version 2.11.3.3) (Waterhouse et al., 2009), employing MAFFT with default settings. Based on the MLRT genotyping results, we selected 10 distinct strains for additional analysis using WGS (**Table 1**). The dendrogram representing genetic variation among 321 isolates of *N. meningitidis* based on MLRT analysis was instrumental in helping us objectively select different strains. Sub-clustering had a major impact on the strain selection process. First, we chose at least two strains (one isolated from blood and one from CSF) from each of the three largest sub-clusters. Secondly, we selected at least two distinct serogroups from each sub-cluster. Additionally, we included rarely identified serogroups (Y, E, NG). We did not choose strains from cluster 1 because we assumed the risk that these strains were from patients with weakened immune systems (patient age >64 years). Additionally, isolates from sub-cluster 3 were recently analyzed by other scientists in Lithuania (Sereikaitė et al., 2023). We did not specifically select for specific restriction types. However, almost all the selected strains had unique restriction types (frequency of 1), with only one strain selected from the most commonly identified restriction type, RT1.

**Table 1:** Characteristics of the 10 selected *Neisseria meningitidis* isolates for whole genome sequencing analysis.

pubMLST ID	Year	Source	Patient age range	Genogroup	MLRT sub-cluster	RT (MLRT)
**120132**	2014	Blood	45-64	C	1	19
**120136**	2016	Cerebrospinal fluid	25-44	B	1	49
**120133**	2015	Blood	15-24	B	2	27
**120134**	2016	Blood	25-44	B	2	43
**120135**	2016	Blood	15-24	E	2	45
**120139**	2017	Cerebrospinal fluid	15-24	Y	2	76
**120140**	2021	Blood	45-64	B	2	83
**119504**	2011	Cerebrospinal fluid	45-64	B	4	1
**120137**	2016	Blood	15-24	NG	4	50
**120138**	2017	Blood	45-64	B	4	58

### 2.5 Bioinformatic analyses

The extracted DNA was sent to Novogene services (https://www.novogene.com/eu-en) for whole genome sequencing using the Illumina NovaSeq 6000 sequencing system and subsequent genome assembly. A paired-end sequencing strategy was used to sequence the samples. The coding genes were predicted using Augustus software (version 2.7) with homologous evidence. The scaffolds were subjected to RAST annotation server (Overbeek et al., 2014). Draft genomes of the described *N. meningitidis* strains were submitted to the PubMLST database (Jolley et al., 2018) with the following accession numbers: 119504, 120132, 120133, 120134, 120135, 120136, 120137, 120138, 120139, and 120140.

Phylogenetic networks were created for the invasive isolates of *N. meningitidis* from Lithuania (n = 43), which were the only genomes available in the PubMLST (Jolley et al., 2018) database until October 9, 2023. Of these, 10 genomes were sequenced in our study, and 33 genomes were submitted earlier. iTOL (Letunic and Bork, 2021) was used to construct neighbor-joining trees using concatenated nucleotides of the core-genome MLST (cgMLST) loci. Loci with paralogous hits within isolates were excluded from analysis.

A minimum spanning tree (MST) was created for a collection of 9490 N*. meningitidis* isolates using the GrapeTree tool on the PubMLST website (Jolley et al., 2018). The tree was constructed based on a cgMLST v2 scheme and was color-coded according to the country of isolation. The analysis specifically included invasive isolates collected in Europe.

tRNA, rRNA, sRNAs, Interspersed Repeat, and Tandem Repeat were predicted by Novogene.

The predicted protein-coding genes were mapped to the Kyoto Encyclopedia of Genes and Genomes (KEGG) using BLASTKOALA and the KEGG Mapper Reconstruction web server (Kanehisa et al., 2016).

The subcellular localization of the proteins was predicted using PSORTb version 3.0 (Yu et al., 2010). Putative adhesion domains in the encoded amino acid sequences were determined using the Pfam database (Mistry et al., 2021). We conducted a literature review to identify experimentally validated bacterial adhesins, such as performing a keyword search for “adhesin,” “adherence,” “adhesion,” “adhesive,” “chitin binding,” “collagen binding,” “laminin binding,” “mucin binding,” “membrane adhesin,” “Neisseria adhesin,” “pilin,” “pilus,” “autotransporter,” “polysaccharide capsule,” “porin,” and “two-partner secretion” in the InterPro 96.0 database (Paysan-Lafosse et al., 2023).

To verify the pathogenicity and virulence factors, PathogenFinder v1.1 (Cosentino et al., 2013) and the Virulence Factor Database (VFDB) (Liu et al., 2019) were used. Antimicrobial resistance genes were identified using Resistance Gene Identifier from CARD (Alcock et al., 2020), and alleles linked to reduced antibiotic susceptibility were detected using the genome comparator tool in PubMLST (Jolley et al., 2018).

## 3 Results

### 3.1 *N. meningitidis* serogrouping

The 321 N*. meningitidis* isolates were serogrouped, and the distribution of serogroups determined by PCR methods was as follows: 292 (91.0%) were serogroup B, 23 (7.2%) were serogroup C, 3 (0.9%) were serogroup Y, 1 (0.3%) was serogroup W135, 1 (0.3%) was serogroup E, and 1 (0.3%) was non-groupable (**Supplementary Table S1**).

### 3.2 Antibiotic susceptibility testing

All the *N. meningitidis* isolates were susceptible to penicillin, cefotaxime, rifampicin, and ciprofloxacin (**Supplementary Table S1**).

### 3.3 *N. meningitidis* genetic diversity

#### 3.3.1 MLRT analysis

**Figure 1:**
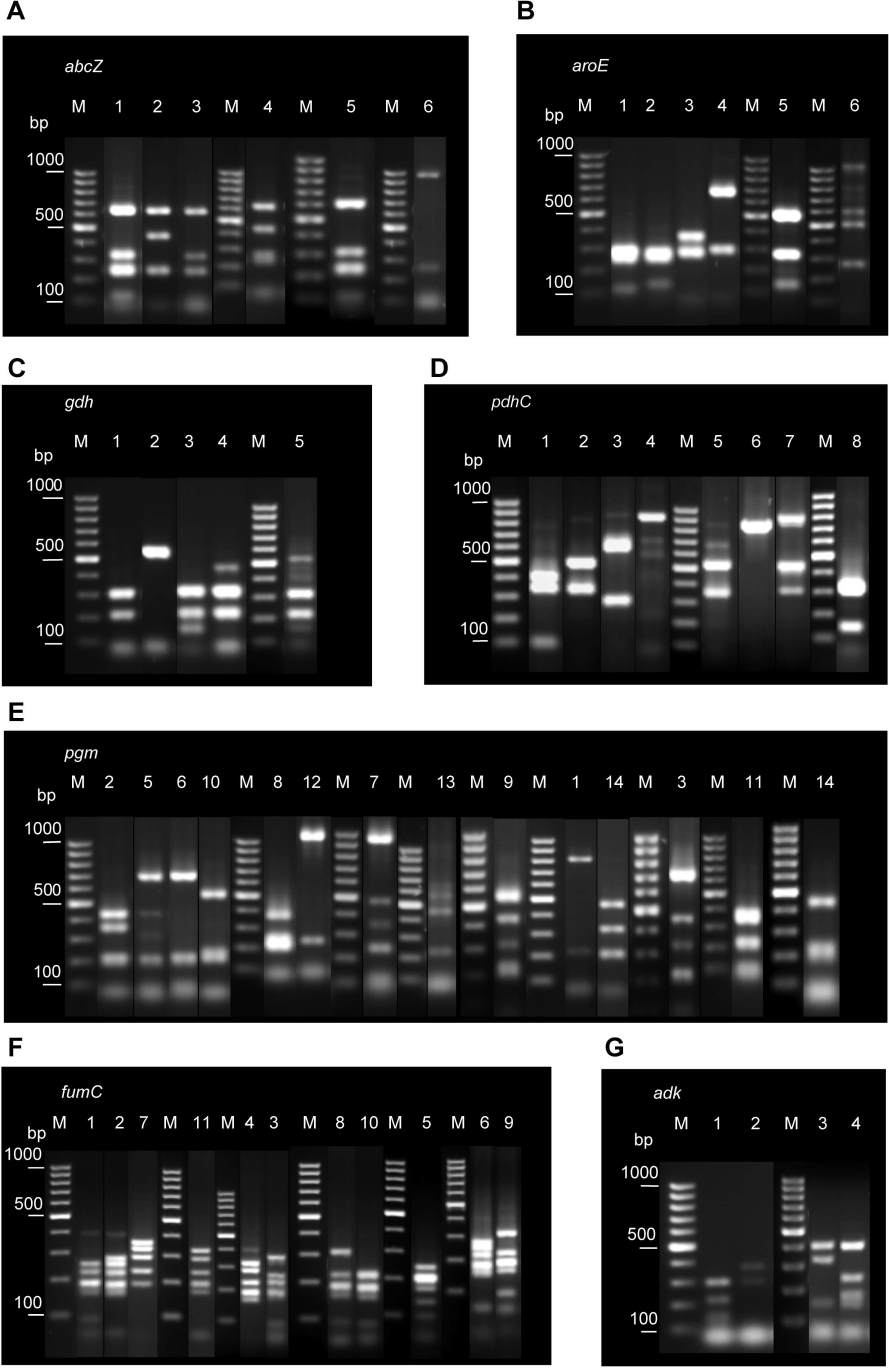
Examples of different restriction patterns obtained following *Msp*I or *Mnl*I digestion of PCR-amplified regions of the seven housekeeping genes analyzed: *abcZ* **(A)**, *aroE* **(B)**, *gdh* **(C)**, *pdhC* **(D)**, *pgm***(E)**, *fumC* **(F)**, and *adk* **(G)**. Lane numbers correspond to allele codes. Lane M, GeneRuler 100 bp DNA Ladder (Thermo Fisher Scientific).

Several restriction patterns (alleles) were observed for each of the seven loci examined, indicating polymorphisms at these loci (**Figure 1**). The number of alleles varied between four in *adk*, the most conserved locus, and 14 in *pgm*, suggesting different evolution rates for different loci.

**Figure 2:**
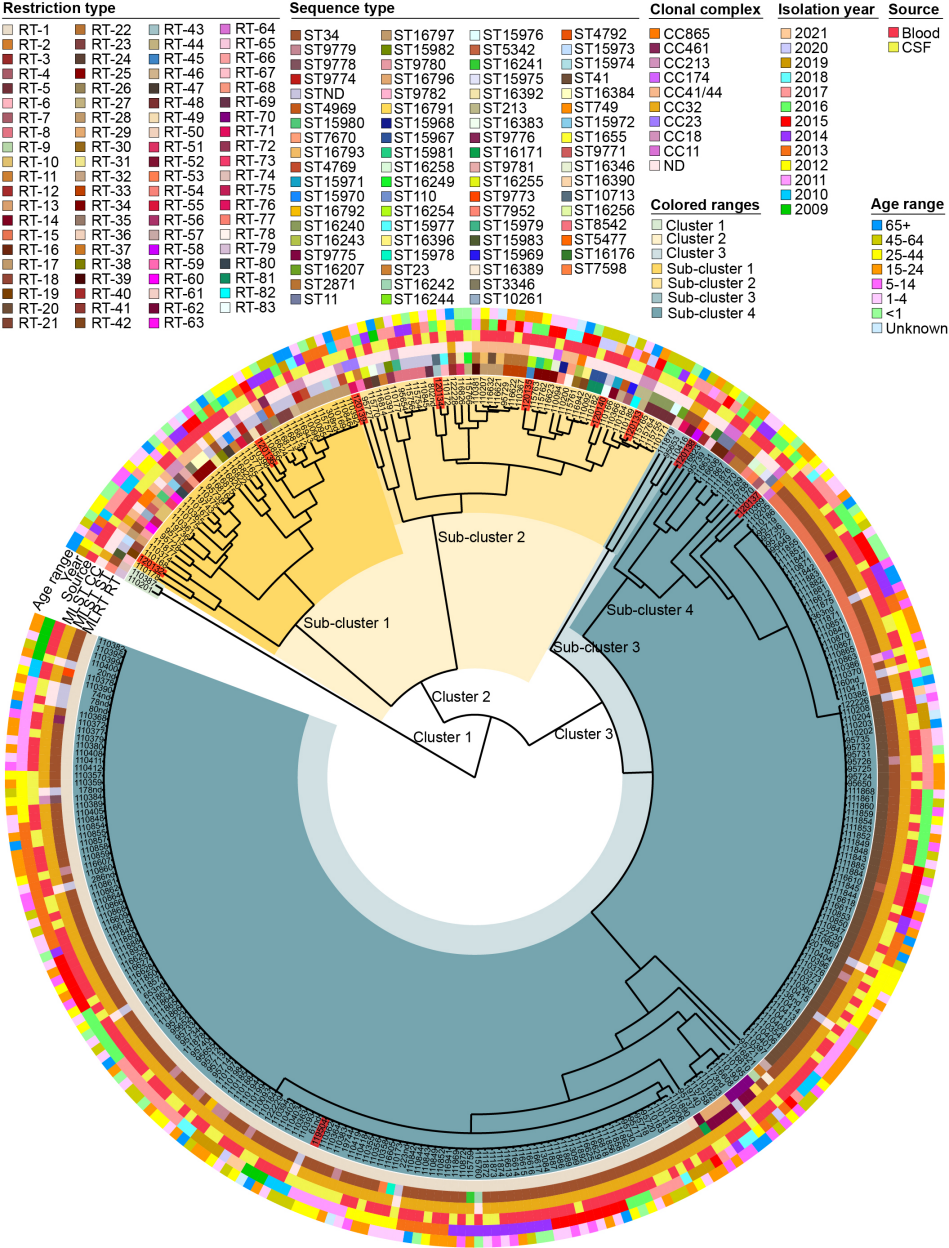
Dendrogram representing genetic variation among 321 isolates of *Neisseria meningitidis* based on MLRT analysis. The isolates highlighted in red are the isolates for which sequence analysis was performed in this study.

Of the 321 strains examined, 83 different RTs were identified; their interrelationships are illustrated in the dendrogram (**Figure 2**). The UPGMA dendrogram illustrated genetic variation among the isolates, categorizing them into three major clusters. Cluster 1 consisted of only two isolates. Clusters 2 and 3 were further divided into two subclusters. Subclusters 1, 2, 3, and 4 were again divided into groups. The first, second, third, and fourth groups included 4, 33, 4, and 39 isolates, respectively. The fifth group comprised only 1 isolate. The sixth group comprised 2 isolates, the seventh group consisted of 93 isolates, and the eighth group included the largest number of isolates (143). Phylogenetic analyses of the isolates revealed that each subcluster consisted of *N. meningitidis* isolates from various isolation years, sites, and patient age groups (**Figure 2**). No significant patterns related to the isolation year, site, or patient age group could be inferred from this clustering. Based on the MLRT genotyping results, we chose 10 distinct strains for molecular characterization using WGS to elucidate evolutionary dynamics, assess regional prevalence, and classify strains more precisely, while also identifying genetic markers and inferring transmission pathways to gain comprehensive insights into emerging strains for enhanced public health understanding and preparedness. The dendrogram was instrumental in helping us objectively select different strains. Sub-clustering had a major impact on the strain selection process, providing additional context and validation for our choices.

To evaluate the relationship between MLRT and MLST genotyping methods, we analyzed data from 10 sequenced isolates and 33 publicly available isolates from Lithuania in the pubMLST database. The distribution of RT, ST, and CC is outlined in the **Supplementary Table S2**. We identified some discrepancies between the identified RTs and STs. The strains marked as RT1, RT15, and RT20 were identified as ST34 according to the MLST method. RT15 differs from RT1 by one allele in two loci, *adk* and *gdh*. RT20 differs from RT1 by one allele in the *gdh* locus.

Although there is some discrepancy between the identified RTs and STs, the clonal relationships among *N. meningitidis* strains are clearly delineated. In our MLRT and MLST comparison analysis, we identified that all strains marked as RT1, RT15, RT20, RT29, and RT70 belonged to the ST-32 complex. According to the MLRT genotyping method, all these strains have identical alleles in the *aroE*, *fumC*, *phdC*, and *pgm* loci. According to the MLST method, all strains belonging to the ST-32 complex have identical alleles in the *adk*, *fumC*, *pdhC*, and *pgm* loci. It is noteworthy that during the visual assessment of electrophoresis, there were doubts in distinguishing allele 2 from allele 3 in the *adk* locus, which might be the cause of the discrepancies.

The strain marked as RT50 was associated with the ST-11 complex, while RT19 and RT49 belonged to the ST-18 complex. Strains marked as RT27 and RT82 were found to be part of the ST-213 complex, and RT76 belonged to the ST-23 complex. Additionally, RT28, RT77, and RT78 were classified within the ST-41/44 complex. Notably, the strain marked as RT17 was the only strain to belong to two distinct clonal complexes: ST-18 (n=2, 66.7%) and ST-32 (n=1, 33.3%).

Our analysis revealed that certain RTs belonged to specific clonal complexes, suggesting that MLRT can serve as a predictor for MLST clonal complexes in our dataset.

#### 3.3.2 *penA* analysis

**Figure 3:**
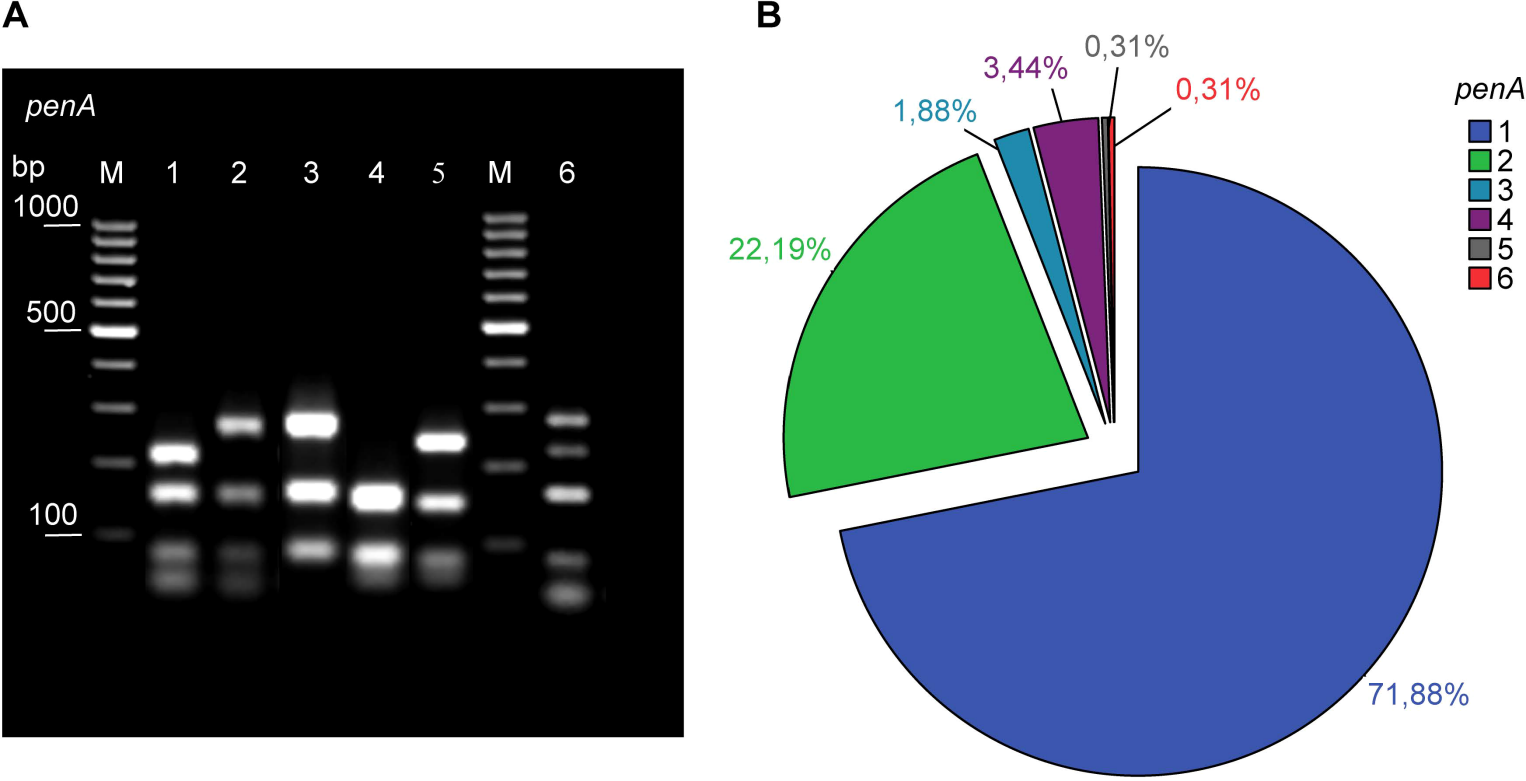
**(A)** Examples of distinct restriction patterns obtained after *Taq*I digestion of the PCR-amplified segment of the *penA* gene, which encodes penicillin-binding protein 2. Lane numbers are associated with allele codes. Lane M, GeneRuler 100 bp DNA Ladder. **(B)** Distribution of distinct restriction patterns obtained after *Taq*I digestion of the PCR-amplified segment of the *penA* gene.

Amplification of the *penA* gene and RFLP analysis were performed to identify the modified *penA* genes. Amplification products were obtained for all analyzed strains. Subsequent digestion with *Taq*I and separation on a 1% agarose gel resulted in the identification of six distinct profiles (**Figure 3A**). A unique identifier was assigned to each profile and labeled patterns 1–6. Among them, *penA* 1 (71.88%, n = 230) was the most prevalent, followed by *penA* 2 (22.19%, n = 71). The distribution of different *penA* restriction patterns in the Lithuanian *N. meningitidis* strains is shown in **Figure 3B**.

The penicillin MIC values in isolates with *penA* 1 (range: 0.016–0.250 mg/L, median = 0.125) were significantly higher than those in isolates with *penA* 2 (range: 0.016–0.190 mg/L, median = 0.023) and *penA* 4 (range: 0.016–0.047 mg/L, median = 0.023) patterns (p < 0.001) (**Supplementary Table S1**). None of the isolates analyzed were resistant to penicillin.

To evaluate whether the *penA* RFLP method used in this study could help identify different *penA* gene modifications, we compared the results with sequencing data from 10 isolates and included an additional 33 publicly available isolates from Lithuania in the pubMLST database. In total, 11 distinct *penA* gene alleles were identified. The *penA52* allele constituted the majority of the analyzed isolates (55.80%, n=24). Notably, all isolates with the *penA52* allele in our study were identified as having the *penA1* restriction pattern. The distribution of *penA* gene alleles and their comparison with the *penA* RFLP method results are presented in the **Supplementary Table S2**.

#### 3.3.3 cgMLST analysis

**Figure 4:**
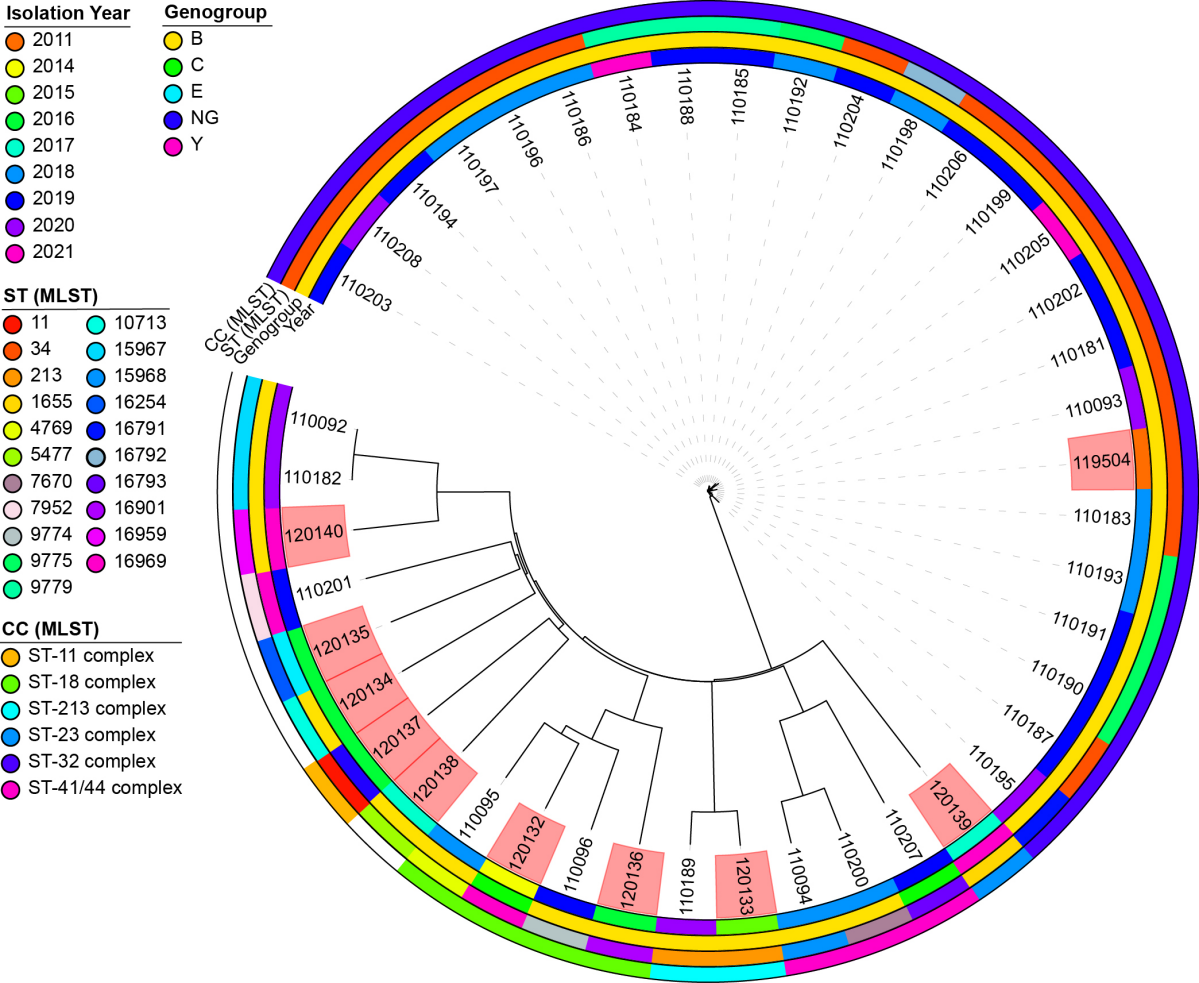
A neighbor-joining tree was constructed based on concatenated cgMLST allele sequences of 43 *Neisseria meningitidis* strains. The isolates are visually represented with color coding, indicating their genogroup, sequence type, clonal complex, and year of isolation. The phylogenetic analysis was performed using the iTOL plug-in available on the PubMLST website (https://pubmlst.org/). The scale bar on the tree represents the genetic distance between the isolates. The isolates highlighted in red are the isolates for which sequence analysis was performed in this study. NG, non-groupable; ST, sequence type; CC, clonal complex.

The genomic diversity of *N. meningitidis* in Lithuania was investigated by analyzing a set of 10 strains specifically examined in this study. To obtain a more thorough and detailed insight into genomic diversity, an additional 33 publicly available genomes collected between 2011 and 2021 were included. All genomes were derived from human sources and associated with invasive infections (**Figure 4**).

Our analysis revealed 21 sequence types (STs) circulating in Lithuania. The most prevalent was ST34 (76.19%, n = 16), followed by ST9775 (19.05%, n = 4) and ST9779 (14.29%, n = 3). Other STs, such as ST213 and ST15967, were detected in at least two genomes, whereas 16 different STs were observed in individual genomes.

Notably, three isolates (ID 120132, 120136, and 120140) displayed unique combinations of seven housekeeping genes and were designated as novel STs upon submission to the PubMLST database. Specifically, isolate 120132 had a novel ST16969 profile, isolate 120136 had an ST16901 profile, and isolate 120140 had an ST16959 profile. Isolate 120132 was obtained from the blood of a 47-year-old male, isolate 120136 from the cerebrospinal fluid (CSF) of a 40-year-old female, and isolate 120140 from the blood of a 46-year-old male.

#### 3.3.4 Comparison of local genomic epidemiology with international genomic epidemiology using the cgMLST scheme

**Figure 5:**
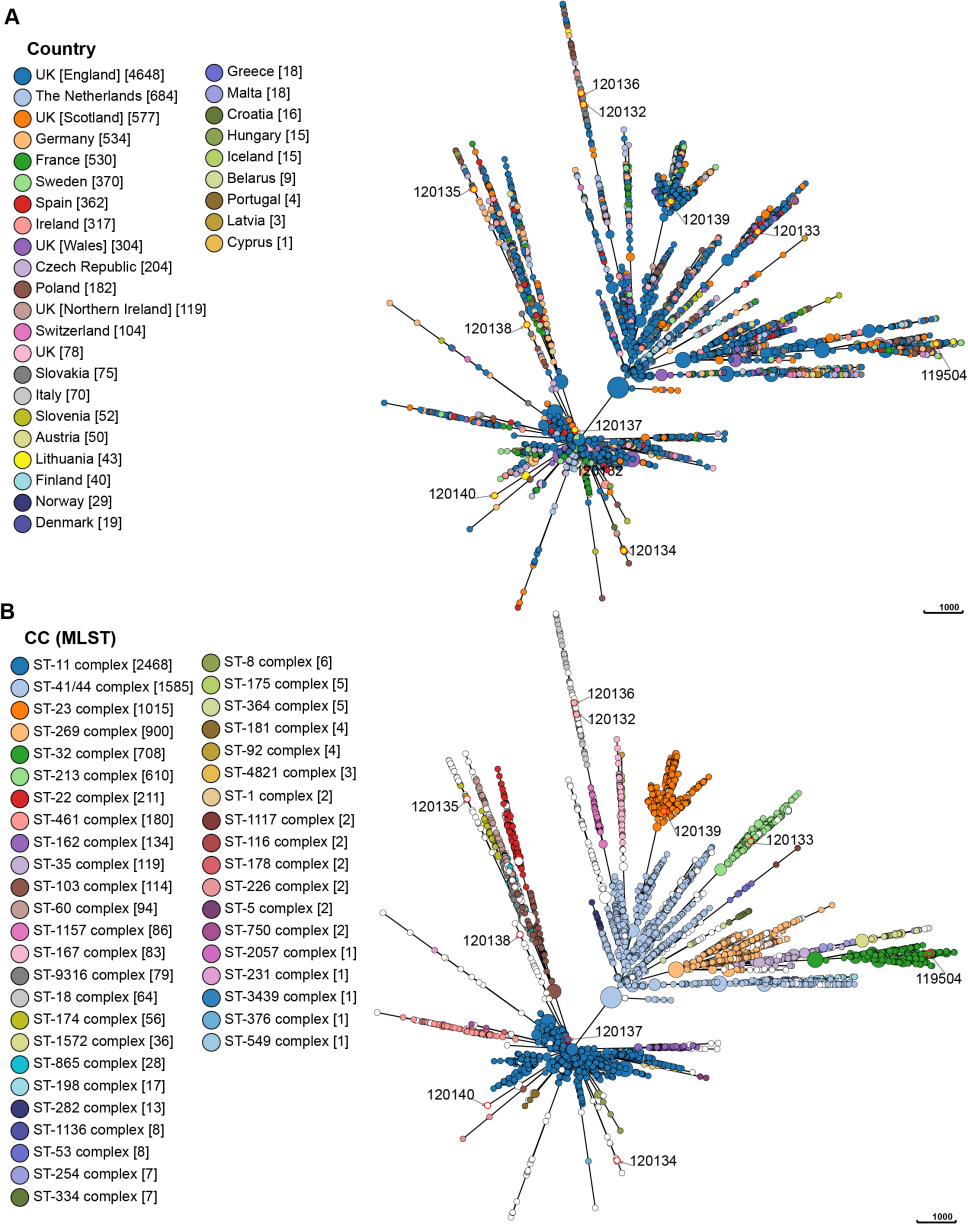
The minimum spanning tree based on the results of cgMLST. Each circle represents genetically related isolates. The length of branches between each node represents the number of different alleles (out of cgMLST genes) that differ between two linked nodes. The size of each circle is proportional to the number of strains it contains. The colors indicate either the corresponding countries of isolation **(A)** or the clonal complex (MLST) **(B)**. The isolates marked with a red circle are the isolates for which sequence analysis was performed in this study. The pubMLST ID number for each isolate for which sequence analysis was performed in this study is written next to each circle.

Incorporating all Lithuanian isolates into a dataset of publicly accessible *N. meningitidis* genomes allowed the evaluation of their genomic diversity and phylogenetic relationships with circulating strains in Europe. Currently, the dataset is predominantly composed of isolates from the United Kingdom (n = 5726), the Netherlands (n = 684), Germany (n = 534), and France (n = 530), contrasting with other European countries, several of which have fewer than 10 sequenced isolates as of the analysis date (e.g., Portugal, Latvia, and Cyprus). Using the cgMLST scheme, the MST of 9490 N*. meningitidis* strains from various European countries revealed no significant geographic relationships. A widespread dissemination of *N. meningitidis* strains was observed within the European region, with the root of the MST originating in Sweden and the United Kingdom. The examined isolates belonged to 43 clonal complexes, with the majority composed of the ST-11 complex (26.0%) and the ST-41/44 complex (16.7%). In Lithuania, however, the ST-32 complex was predominant (58.1%), contrasting with its lower prevalence (7.5%) in the broader European region. Out of the 9490 analyzed isolates, 814 (8.6%) were not assigned to a clonal complex. Of the 10 Lithuanian isolates analyzed in this study, eight belonged to different clades of relatedness, while only two strains (ID 120132 and 120136) were closely related (**Figure 5**).

MST demonstrated that the Lithuanian ST-32 complex isolates (n = 25), including the isolate analyzed in this study (ID 119504), were genetically related and formed a single clade, along with isolates from various European countries. The newly identified STs, ST16969 (ID 120132) and ST16901 (ID 120136), were closely related to an isolate found in England in 2016 (ID 53173) and belongs to ST18. Additionally, several isolates from Lithuania exhibited close genetic relationships with isolates from England, Northern Ireland, Poland, and the Czech Republic, indicating potential transmission events and genetic exchange across borders. **Supplementary Figures S1, S2** illustrate the genetic relationships between *N. meningitidis* strains from Lithuania and those circulating in Europe.

### 3.4 Analysis of WGS data

In this study, ten draft genomes of *N. meningitidis* strains were used. The characteristics of the assembled genome are listed in **Supplementary Table S3**. The number of scaffolds per genome assembly varied from 70 to 121, and the N50 values were between 29,431 and 55,311 base reads. The average genome size of the 10 strains was 2,163,040 bp, ranging from 2,060,116 bp (ID 120137) to 2,283,261 bp (ID 120136). The average GC content of all the genomes was 51.66%, ranging from 51.46% (ID 120138) to 51.95% (ID 120137).

The average number of coding genes predicted by Augustus (version 2.7) was 2178, ranging from 2082 (ID 120137) to 2305 (ID 120136). In contrast, the average number of CDSs by RAST was 2567, ranging from 2429 (ID 120137) to 2729 (ID 120136). Only 28–31% of CDSs in each strain could be functionally categorized into subsystems. In all studied strains, subsystems such as protein metabolism, amino acids and derivatives, cofactors, vitamins, prosthetic groups, and pigments were found to have a higher number of functional genes. No genes associated with potassium metabolism, motility, or chemotaxis were detected in any strain. **Supplementary Table S3** and **Supplementary Figure S3** provide a comprehensive overview of the genome characteristics of *N. meningitidis* strains and their subsystem statistics.

Based on KEGG analysis, the CDSs were categorized into six sub-categories. The strains conserved 1264 (ID 120137) to 1288 (ID 120136) CDSs, which were further classified into 40 functional KEGG subcategories. It is worth mentioning that all strains exhibited a noticeably higher abundance of genes associated with metabolic pathways (CDSs 783–803), followed by genetic information processing (CDSs 183–186) and human diseases (CDSs 106–112) (**Supplementary Table S4**). Pathway modules (**Supplementary Table S5**) showed high similarity across strains, with few differences observed. The exclusive metabolic features detected in strains 120136, 120139, and 120140 were complete pathways for dTDP-L-rhamnose biosynthesis. In other strains, this pathway was either not detected (119504 and 120133) or was incomplete (120132, 120134, 120135, 120137, and 120138). Incomplete pathways for Shikimate and Siroheme biosynthesis were observed in strains 120134 and 120137, respectively.

#### 3.4.1 Genetic factors encoding adhesion properties

Exploration of extracellular proteins or outer membrane-attached proteins encoded within the genomes of the analyzed strains, as indicated by PSORTb, revealed an average of 66 proteins (range: 62–77). Additionally, all genomes were examined for the presence of adhesion-related domains. We identified 50 distinct adhesion-related domains. The subsequent domains were found in all strains: PF03865 and PF13018. The following domains were exclusively detected in strain ID 120139: PF06122, PF05309, PF06834, and PF09673. The results of identifying adhesion-related domains and their frequencies in the analyzed genomes are presented, and a complete list of domains detected using the Pfam database can be found in **Supplementary Tables S6, S7**. **Supplementary Table S8** contains a comprehensive list of proteins found in the genomes of *N. meningitidis* strains, including those located extracellularly, attached to the outer membrane, and putative adhesins in other cellular locations, along with their respective domains, as identified using the Pfam database. From these analyses, we identified genes encoding putative adhesins or adhesion-related proteins. These candidates should be further investigated in future functional studies.

#### 3.4.2 Genome-based assessment of the potential for pathogenicity

The 10 N*. meningitidis* genomes were screened for pathogenicity and virulence genes using the PathogenFinder server, scoring each strain as a potential human pathogen (**Supplementary Table S3**). Strain 120134 had the lowest pathogen score of 85.3%. A total of 1732 genes associated with pathogenicity were identified, with nonuniform distribution across the genomes of each strain. Strains 119504 and 120137 exhibited the highest number of detected pathogenicity determinants (656 and 636, respectively), while strain 120133 had the lowest (350). All 10 isolates were positive for 17 pathogenicity determinants, with five of them categorized as hypothetical proteins. The pathogenic determinants present in a single isolate were defined as unique determinants. In total, 873 unique pathogenicity determinants were identified, 383 of which were designated as hypothetical and 237 as putative proteins. Strain 120134 had the fewest unique determinants (29), while strains 119504 and 120137 had the most at 345 and 227, respectively.

**Figure 6:**
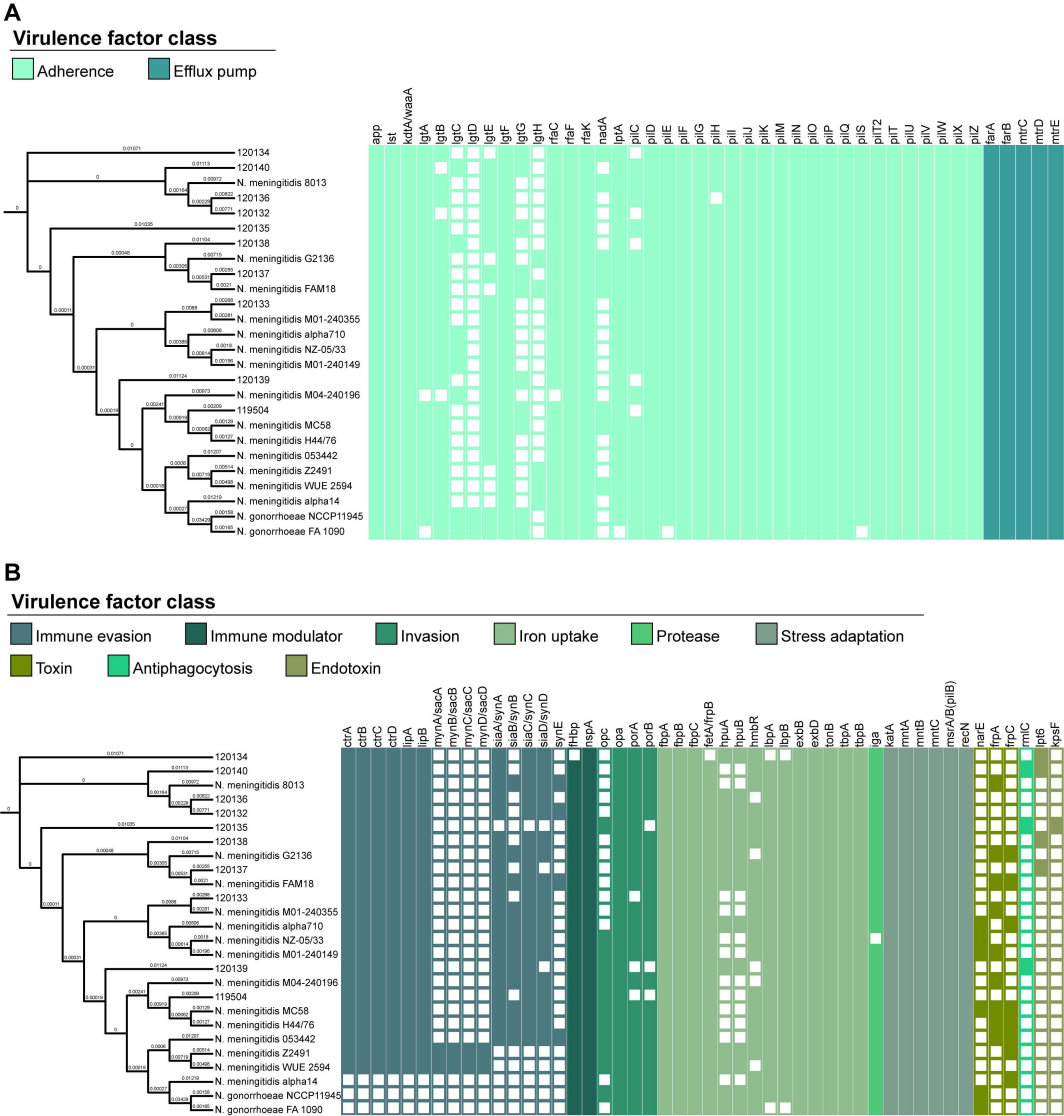
Distribution of virulence genes predicted in *Neisseria meningitidis* isolates collected in Lithuania and in strains of *Neisseria meningitidis* and *Neisseria gonorrhoeae* archived in the Virulence Factor Database. A neighbor-joining tree was constructed based on cgMLST allele sequences of *Neisseria meningitidis*. The presence of virulence genes is depicted by the colored network, whereas their absence is indicated by the empty network. **(A)** Genes associated with adherence and efflux pump. **(B)** Genes associated with immune evasion, immune modulator, invasion, iron uptake, protease, stress adaptation, toxin, antiphagocytosis, and endotoxin.

The VFDB system confirmed the presence of various virulence genes, categorized into 11 classes, with most genes associated with adherence (46.3%) and iron uptake (17.1%) (**Figure 6**). Among the identified genes, 57 (69.5%) were present in all analyzed strains. Notably, genes related to antiphagocytosis (*rmlC*) and endotoxins (*lpt6* and *kpsF*) were exclusively found in Lithuanian isolates analyzed in this study. Isolates 120134 and 120135 lacked the *fetA*/*frpB* and *pilH* genes, respectively, in contrast to the other isolates in this study and the strains stored in the database. **Supplementary Table S9** provides a comprehensive overview of the virulence factor genes of *the N. meningitidis* strains analyzed in this study.

#### 3.4.3 Antibiotic resistance

For all the strains examined, at least one gene or gene mutation leading to resistance to antimicrobial agents was identified. Eight antibiotic resistance genes have been detected that confer resistance to antibacterial free fatty acids, macrolides, cephalosporins, cephamycin, tetracycline, penicillin, disinfecting agents, and antiseptics. Overall, the most common resistance genes detected were *farB* (90.0%), which mediates resistance to long-chain antibacterial fatty acids, and *mtrA* (100%), a macrolide antibiotic. The mutation V57M in *rpsJ*, conferring tetracycline resistance, was observed in strain 120139. However, these factors are not advantageous because they are not the antibiotics of choice for the treatment of IMD. Three strains harbored mutations in *N. meningitidis* PBP2, conferring resistance to beta-lactam. A complete list of resistance genes detected using the CARD database is provided in **Supplementary Table S10**.

We identified allele diversity at all loci among the 11 antibiotic susceptibility genes analyzed using PubMLST. Three isolates harbored alterations in *penA*, encoding penicillin-binding protein 2, with specific substitutions: isolate ID 119504 had the *penA52* allele, isolate ID 120136 had *penA686*, and isolate ID 120138 had *penA1191*. In the PubMLST database, only two isolates were found to have the *penA686* allele: one from Lithuania and one from Italy (ID 40340), while the *penA1191* allele was identified exclusively in the isolates examined in this study. Next, we used BLASTn to detect homologies between rarely detected *penA* alleles (*penA686* and *penA1191*) and *penA* genes from other *Neisseria* species. A BLASTn search against the NCBI nr database yielded hits in other *Neisseria* species with high (>95%) sequence identity. One of the rarest alleles (*penA1191*) showed an identity score of 96.51% with the *penA* gene sequences of *N. gonorrhoeae* strain NGMAST ST7268 (GenBank accession no. KC192769.1) and *N. gonorrhoeae* strain 31188 (GenBank accession no. JF893455.1). In the *penA686* nucleotide sequence analysis, we found matches with 95.08% identity to the *N. gonorrhoeae* strain NG9901 *penA* gene (GenBank accession no. AB608050.1).

The β-lactamase gene (blaROB-1), associated with resistance against β-lactam antibiotics (Hong et al., 2018), was not detected in any analyzed isolates. No alleles linked to resistance to fluoroquinolones (*gyrA*, NEIS1320, NEIS1525, and NEIS1600) or rifampicin (NEIS0123 and *rpoB*) were found (**Table 2**).

**Table 2:** Allelic profiles of antibiotic resistance genes in Neisseria meningitidis isolates from Lithuania.

Locus	pubMLST ID									
	119504	120132	120133	120134	120135	120136	120137	120138	120139	120140
*gyrA*	2	4	3	4	12	11	4	4	1	3
NEIS0123	132	41	39	302	218	41	1	2909	39	2382
NEIS0414	4	1	22	244	168	207	1	1	45	X
NEIS1320	2	2216	97	1	174	2217	159	9	107	2218
NEIS1525	51	50	49	623	2155	2156	44	224	70	2627
NEIS1600	22	260	57	289	1570	1571	172	202	79	1572
NEIS1635	4	7	7	9	11	7	1	2	7	7
NEIS1753	189	2643	70	205	211	3498	59	2644	8	9
NEIS3240	X	X	X	X	X	X	X	X	X	X
*penA*	52	5	34	1	5	686	1	1191	22	4
*rpoB*	2	1	34	34	5	1	9	X	34	34

Each locus was assigned an allele number according to its DNA sequence using the PubMLST database.

X – missing alleles.

##### 3.4.3.1 Alterations of PBP2

**Figure 7:**
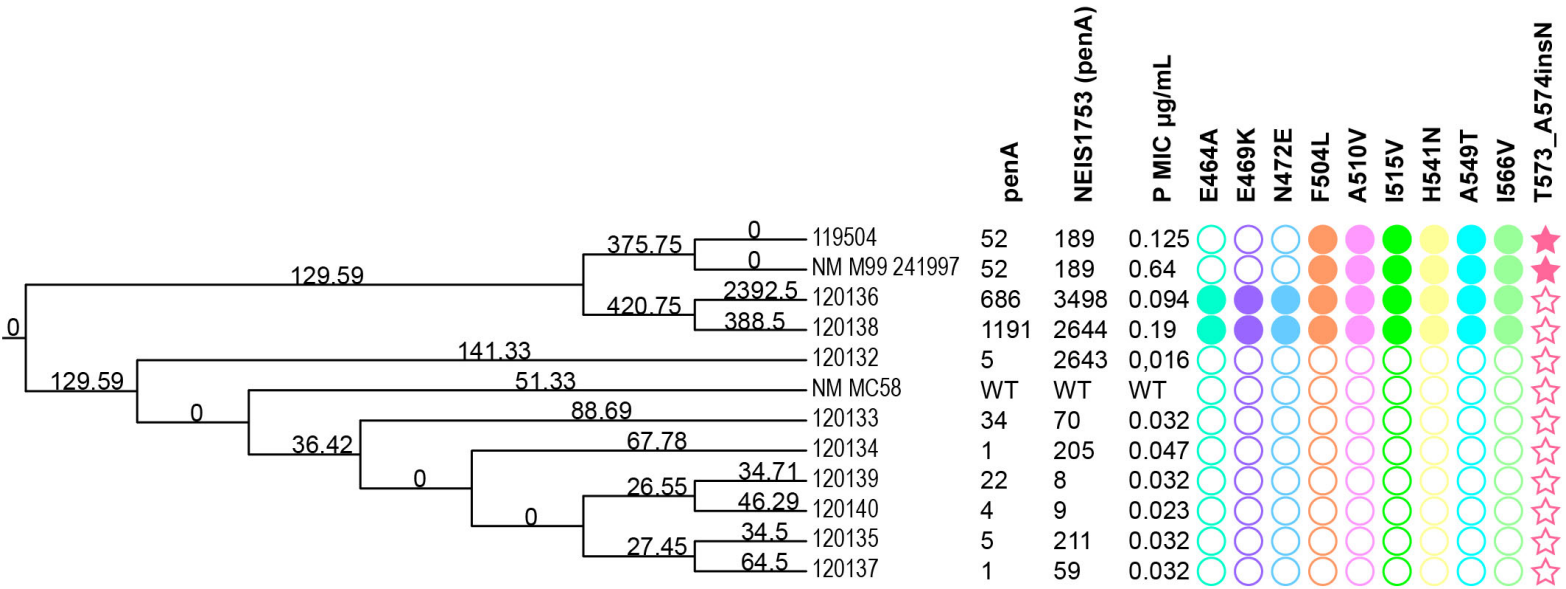
Neighbor-joining phylogenetic tree based on the NEIS1753 (penA) locus SNPs of 10 N*. meningitidis* isolates from Lithuania, the penicillin-resistant *N. meningitidis* strain M99 241997, and the penicillin-sensitive reference strain *N. meningitidis* MC58. The tree includes annotations for *penA* and NEIS1753 alleles extracted from the pubMLST database, penicillin MIC values, and amino acid mutations associated with penicillin resistance. WT – wild type penicillin-sensitive. Filled shapes indicate the presence of mutations, and empty shapes indicate the absence of mutations. Circles represent amino acid substitutions, while stars represent amino acid insertions.

The complete nucleotide sequences of the NEIS1753 (*penA*) locus from 10 analyzed isolates were examined to identify alterations in *penA* gene. These sequences were compared with the *N. meningitidis* MC58 strain (GenBank accession no. AE002098.2), which harbors the wild-type *penA* gene, and with the *N. meningitidis* M99 241997 strain extracted from pubMLST database (ID 89367) and which is resistant to penicillin (MIC=0.64 μg/mL) (**Figure 7**; **Supplementary Figure S4**). Three isolates harbored different mosaic *penA* alleles (*penA52*, *penA686*, and *penA1191*). The sequences of the divergent mosaic alleles were 89.55% to 97.94% identical to the wild-type sequence represented by the MC58 strain. Specifically, isolate 119504 showed 97.94% identity, isolate 120136 showed 89.55% identity, and isolate 120138 showed 96.19% identity. This comparison was based on the complete nucleotide sequences of the NEIS1753 (*penA*) locus. Isolates 119504 and 120138 have slightly increased penicillin resistance (MICs of 0.125 μg/mL and 0.19 μg/mL, respectively).

In the *penA* C-terminal domain of PBP2 (amino acids 298 to 581), the amino acid sequences were aligned. There are 12 amino acid differences between NmMC58 and Lithuanian isolate ID 119504, 24 differences between NmMC58 and isolate ID 120136, and 14 differences between NmMC58 and isolate ID 120138 (**Supplementary Figure S4**). All three isolates possessed six penicillin-resistance-associated mutations (F504L, A510V, I515V, H541N, A549T, and I566V). These mutations are also found in the penicillin-resistant strain M99 241997. Additionally, isolates ID 120136 and ID 120138 possessed the E464A, E469K, and N472E substitutions, which were not found in the M99 241997 strain. All these amino acid sites have been previously linked to decreased susceptibility to beta-lactam antibiotics.

Lithuanian isolate ID 119504 and the penicillin-resistant strain M99 241997 have an insertion of a single asparagine amino acid between Thr573 and Ala574 (T573_A574insN) along with a substitution of one amino acid downstream (A574V). The sequences of the *penA* genes of isolates ID 120132, 120133, 120134, 120135, 120137, 120139, and 120140 were identical to that of the *N. meningitidis* MC58 strain.

## 4 Discussion

This study aimed to characterize the isolates of *N. meningitidis* causing IMD collected in Lithuania from 2009 to 2021. Various techniques were used, including MLRT genotyping, *penA* gene RFLP analysis, phylogenetic group detection, and WGS analysis.

The epidemiology of IMD has undergone significant changes due to factors like vaccination efforts, environmental conditions, ease of travel, and mass gatherings (Acevedo et al., 2019). Although Europe has seen an increase in serogroup W meningococcus (MenW) and MenY cases (Zografaki et al., 2023), MenB remains the major cause of IMD in Lithuania, with no recent rise in MenW and MenY cases. A rapid decrease in the number of IMD cases was observed shortly after the initiation of the initial lockdown in numerous countries during the COVID-19 pandemic (Brueggemann et al., 2021). A decline in IMD cases was observed in Lithuania starting in 2018, before the COVID-19 pandemic, reflecting the impact of vaccination efforts, including the introduction of the 4CMenB vaccine (Bexsero) into the national immunization program in July 2018. In recent studies conducted in Lithuania, the MenDeVAR Index method (Rodrigues et al., 2021) was used to estimate the predicted coverage of the 4CMenB and MenB-FHbp vaccines. The findings concluded that both serogroup B vaccines demonstrate potential to protect against IMD in Lithuania (Sereikaitė et al., 2023). Importantly, this study analyzed isolates recovered in Lithuania from 2009 to 2019, including the period before vaccination and shortly after the implementation of the 4CMenB vaccine (Bexsero) into the national immunization program in July 2018. Therefore, it is crucial to determine the MenDeVAR index and conduct a comprehensive characterization of *N. meningitidis* in the post-vaccination period, which would provide valuable insights for public health strategies.

Studies suggest that diverse regional surveillance systems and the evolution of hypervirulent *N. meningitidis* strains pose a potential threat, potentially increasing IMD incidence (Acevedo et al., 2019). For effective meningococcal disease surveillance, high-resolution, comprehensive, and portable typing schemes are crucial (Jolley et al., 2007). In this study, MLRT method (Bennett and Cafferkey, 2003) was used to characterize *N. meningitidis* strains, revealing polymorphisms at the examined loci. MLRT genotyping of 321 isolates from Lithuania identified 83 strains, highlighting MLRT’s discriminatory, rapid, and cost-effective nature for *N. meningitidis* strain characterization. These findings support previous studies characterizing *N. meningitidis* strains (Bennett et al., 2020). The population of *N. meningitidis* isolates in Lithuania displayed heterogeneity, consistent with global isolate diversity trends (Caugant and Brynildsrud, 2019). Our results indicate that MLRT method can assist in identifying clonal relationships among *N. meningitidis* strains associated with meningococcal disease, without the need for expensive equipment or a high level of technical expertise to perform. This simple and cost-effective method can be used as a screening tool to quickly identify newly emerging *N. meningitidis* strains. It helps in objectively selecting new strains for further analysis using more sensitive techniques like whole-genome sequencing. This approach is particularly useful in countries where advanced methods like MLST genotyping or WGS are not routinely used for epidemiological surveillance.

Treatment options for confirmed IMD typically involve third-generation cephalosporins, penicillin when the strain is susceptible, or chloramphenicol for beta-lactam antibiotic allergies (Nadel, 2016). Recently, strains of *N. meningitidis* resistant to antibiotics recommended for treatment and chemoprophylaxis have been detected globally (Rostamian et al., 2022). Modifications to penicillin-binding protein 2 reduce its affinity for penicillin, resulting in decreased susceptibility (Acevedo et al., 2019). In this study, we identified six distinct *penA* profiles using amplification of the *penA* gene and RFLP analysis, providing a cost-effective method for investigating prevalent *penA* modifications.

In previous research, geographic variation in the distribution of different *penA* alleles has been observed (Taha et al., 2007). In 2016, *N. meningitidis* serogroup B ST-32 complex, which harbored the *penA52* allele, was associated with a clonal epidemic of IMD in France (Thabuis et al., 2018). Isolates in Lithuania predominantly harbored the *penA52* allele, which this study unequivocally assigned to the *penA1* restriction pattern. We found that isolates with the *penA1* restriction pattern have a statistically significantly higher penicillin G MIC. Other studies have also observed higher penicillin MIC in strains with the *penA52* allele (Hedberg et al., 2009). Notably, pubMLST provides data on the penicillin susceptibility of 72 strains with the *penA52* allele. Of these, 19 strains (26.4%) are resistant to penicillin, with penicillin concentrations ranging from 0.064 mg/L to 0.640 mg/L (https://pubmlst.org/).

The meningococcal population structure is diverse and dynamic, with distinct strains grouped into various clonal complexes, some of which are associated with IMD (Caugant and Maiden, 2009). In Lithuania, genomic diversity analysis revealed 21 different circulating STs, with ST34 being the most prevalent (76.19%). Three isolates (ID 120132, 120136, and 120140) displayed unique combinations of housekeeping genes and were identified as novel STs (ST16969, ST16901, and ST16959). Importantly, these isolates with newly identified STs were obtained from adult individuals (ages 40-47 years). It is noteworthy that IMD primarily affects infants in most countries (European Centre for Disease Prevention and Control, 2023).

Using the cgMLST scheme, MST revealed no significant geographic relationships. The ST-32 complex was most prevalent in Lithuania (58.1%) but less so in other European countries (7.9%). Lithuanian isolates in the ST-32 complex formed a distinct clade with isolates from the United Kingdom, Netherlands, Germany, France, Belarus, and Poland. Notably, the most prevalent clonal complex in Lithuania, CC32, is associated with hyperinvasive strains and global outbreaks*. N. meningitidis* CC32 caused outbreaks in the United Kingdom during the 1980s (Mikucki et al., 2022) and in the United States from 2013 to 2017 (Soeters et al., 2019). This study provides valuable insights into the epidemiological characteristics of IMD in Lithuania. To the best of our knowledge, this is the first study to investigate the genotypic relationship between Lithuanian isolates and strains isolated in Europe with the aim of determining the relatedness of Lithuanian isolates based on core-genome analysis.

Over the last decade, WGS has emerged as a leading technique in clinical microbiology (Purushothaman et al., 2022). The genomes of 10 distinct *N. meningitidis* strains were analyzed using the advancements offered by WGS to gain a better understanding of the biology of *N. meningitidis* and the epidemiology of IMD in Lithuania. Initially, we observed that the analyzed strains harbored a genome of approximately 2.2 million bases and a GC content of 51.7%, as previously described elsewhere (Caugant and Brynildsrud, 2019). KEGG analysis revealed highly similar pathway modules among the strains, with only minor differences observed. We found that two *N. meningitidis* strains displayed incomplete pathways for Shikimate and Siroheme biosynthesis, potentially affecting their metabolic capabilities and nutritional requirements. Additionally, only three strains possessed a complete pathway for dTDP-L-rhamnose biosynthesis. dTDP-L-rhamnose is crucial for building cell surface structures essential for bacterial interactions with the environment, including the host immune system (van der Beek et al., 2019). Further investigation of the specific genetic differences associated with this pathway in strains with complete and incomplete pathways could provide insights into the molecular basis of these variations and their potential impact on the biology, adaptation, and pathogenicity of *N. meningitidis*.

The initial bacterium-host interaction relies on adhesion, where specific bacterial surface molecules (adhesins) bind to host cell receptors. Various surface structures like pilus, opacity-associated proteins (Opa and Opc), autotransporter adhesins (App, MspA/AusI, NadA, and NhhA), two-partner secretion systems (HrpA and HrpB) play key roles. Additionally, other surface structures like polysaccharide capsules, lipo-oligosaccharides, and porins also contribute to adhesion (Hung and Christodoulides, 2013). Our results revealed combinations of adhesion domains and possibly adhesion-related domains in *N. meningitidis* isolates from Lithuania.

Previous studies have indicated that approximately 17% of *N. meningitidis* strains possess a Gonococcal Genetic Island (GGI). However, some strains exhibit multiple insertions and deletions within the GGI, whereas others harbor intact T4SS genes, potentially leading to the production of functional secretion systems (Woodhams et al., 2012). During the domain analysis, we identified a strain with a unique combination of domains that was not found in the other strains examined. The domains identified in strain ID 120139 (TraL, TraE, TraK, TraC, TraU, type-F conjugative transfer system pilin assembly protein, F plasmid transfer operon protein, TraG-like protein, and N-terminal region) were encoded by GGI. Although it has been suggested that GGI is absent in serogroups A, B, and Y (Woodhams et al., 2012), our study found specific GGI sequences in the *N. meningitidis* serogroup Y.

The analyzed isolates possessed the essential virulence factors required for effective colonization and pathogenicity, affirming their disease-causing potential. The outer membrane vesicles, NhhA, and the capsules are among the most widely recognized and extensively studied virulence factors of *N. meningitidis* (Joshi and Saroj, 2023). According to the VFDB, the analyzed strains possessed virulence factors not commonly found in *N. meningitidis*, such as types of endotoxins (LOS) found in the genus *Haemophilus* and a capsule-associated virulence factor for antiphagocytosis found in the genus *Vibrio*. In this study, 82 genes linked to virulence were identified, most of which were associated with adherence and iron uptake, which is consistent with the well-established dependence of *N. meningitidis* growth on iron availability.

In addition to virulence factors, the analyzed isolates contained various antibiotic-resistance genes, providing resistance to multiple classes of antibiotics. *penA* gene mosaic alleles, characterized by four or five amino acid substitutions, have been shown to induce intermediate susceptibility to penicillins (Taha et al., 2007). Three isolates in our study harbored alterations in *penA*, encoding penicillin-binding protein 2, carrying E464A, E469K, N472E, F504L, A510V, I515V, H541N, A549T and I566V substitutions. These amino acid sites have been previously linked to decreased susceptibility to beta-lactam antibiotics, such as penicillin and cephalosporins (Hao et al., 2011). The isolate with the *penA52* allele, for which the penicillin MIC was 0.125 μg/mL, had an asparagine insertion T573_A574insN in its *penA* gene. The significance of the Asn insertion in PBP2 is unclear (Du Plessis et al., 2008). To investigate the association between the asparagine insertion and penicillin susceptibility, we analyzed *penA* gene sequences from 209 N*. meningitidis* isolates with penicillin MICs above 0.25 μg/mL and 137 isolates with penicillin MICs equal to or below 0.016 μg/mL, sourced from the pubMLST database. Our findings indicate that 70% of the penicillin-resistant isolates contained an additional asparagine (AAT) codon in their *penA* genes. In contrast, none of the penicillin-susceptible isolates possessed this insertion (data not shown).

Another mechanism leading to penicillin resistance is the presence of *bla* genes that encode beta-lactamases (Tsang et al., 2019). The blaROB-1 gene was not present in any of the analyzed isolates. Studies have shown that resistance to ciprofloxacin can be induced by mutations in *parC* or the quinolone resistance-determining region of *gyrA* (Chen et al., 2020), while resistance to chemoprophylactic agents, such as rifampicin, due to mutations in *rpoB* has also been documented (Taha et al., 2010). No alleles were associated with phenotypic resistance to fluoroquinolones (*gyrA*, NEIS1320, NEIS1525, and NEIS1600) or rifampicin (NEIS0123 and *rpoB*). In the current study, we identified *N. meningitidis* strains with novel *penA*, NEIS0123, NEIS1320, NEIS1525, NEIS1600, and NEIS1753 locus variants. However, further exploration of antibacterial resistance using phenotypic and transcriptomic analyses is necessary to obtain a comprehensive understanding of how newly detected gene alleles correlate with antimicrobial sensitivity.

While *N. meningitidis* remains susceptible to antibiotics employed for the treatment and prevention of IMD, concerns persist regarding reduced antibiotic susceptibility (Rostamian et al., 2022). In our study, none of the analyzed isolates exhibited phenotypic resistance to penicillin, cefotaxime, rifampicin, or ciprofloxacin. However, the identification of several strains with genes associated with antimicrobial resistance raises the concern that resistance may emerge in the future, potentially altering the epidemiology of IMD in Lithuania.

In summary, this study evaluates IMD trends in Lithuania from 2009 to 2021. However, it has limitations. Firstly, the relatively small number of available genomes for WGS analysis may have limited the detection of unique genetic characteristics specific to strains circulating in Lithuania. Secondly, the data collected from a limited number of sources did not reflect the prevalence of *N. meningitidis* among carriers. Another limitation of this study relates to our genome assemblies being based on short-read sequencing only. This may introduce the possibility that some genes were not detected due to the incompleteness of the genome. However, our draft genomes exhibited high quality indicators (low contig number, expected genome size, and high coverage), which gives us confidence in our reported results. In future studies, PCR targeting the regions of interest or long-read sequencing technologies such as Nanopore sequencing, capable of generating ultra-long (>4Mb) reads, could be employed to enhance the reconstruction and characterization of genes in *N. meningitidis* isolates. Consequently, it is challenging to determine the distinct characteristics of MenB CC32 genomes in Lithuania, as observed through WGS, which distinguishes them from MenB CC32 and nonvirulent strains found in other regions. This limitation hinders our ability to reliably explain variations in virulence and pathogenicity. Finally, the identified genetic virulence, pathogenicity, and antibiotic resistance determinants have not been experimentally confirmed.

To the best of our knowledge, this is the first study to explore the genetic characteristics of *N. meningitidis* isolates from Lithuania and to investigate the genotypic relationship of Lithuanian isolates with strains isolated in Europe, with the aim of determining the relatedness of Lithuanian isolates based on core-genome analysis. Finally, this study enriches pubMLST database with WGS data from Lithuanian isolates. While Lithuania has not experienced any outbreaks thus far, it is essential to prioritize the molecular epidemiological surveillance of *N. meningitidis* as hypervirulent strains continue to emerge.

## Data Availability

The datasets generated and analyzed in this study have been deposited in the Public Databases for Molecular Typing and Microbial Genome Diversity (PubMLST). The genome sequences are accessible at the following link: https://pubmlst.org/bigsdb?db=pubmlst_neisseria_isolates&page=query&genomes=1, with the accession numbers as follows: 119504, 120132, 120133, 120134, 120135, 120136, 120137, 120138, 120139, 120140.
