# Supplementary material for: Genetic characterization of Neisseria meningitidis isolates recovered from patients with invasive meningococcal disease in Lithuania
Source: Front Cell Infect Microbiol. 2024 Oct 14;14:1432197. doi: 10.3389/fcimb.2024.1432197 (PMC11513629; doi:10.3389/fcimb.2024.1432197)
Supplement: Supplementary file 1 [file DataSheet1.pdf]

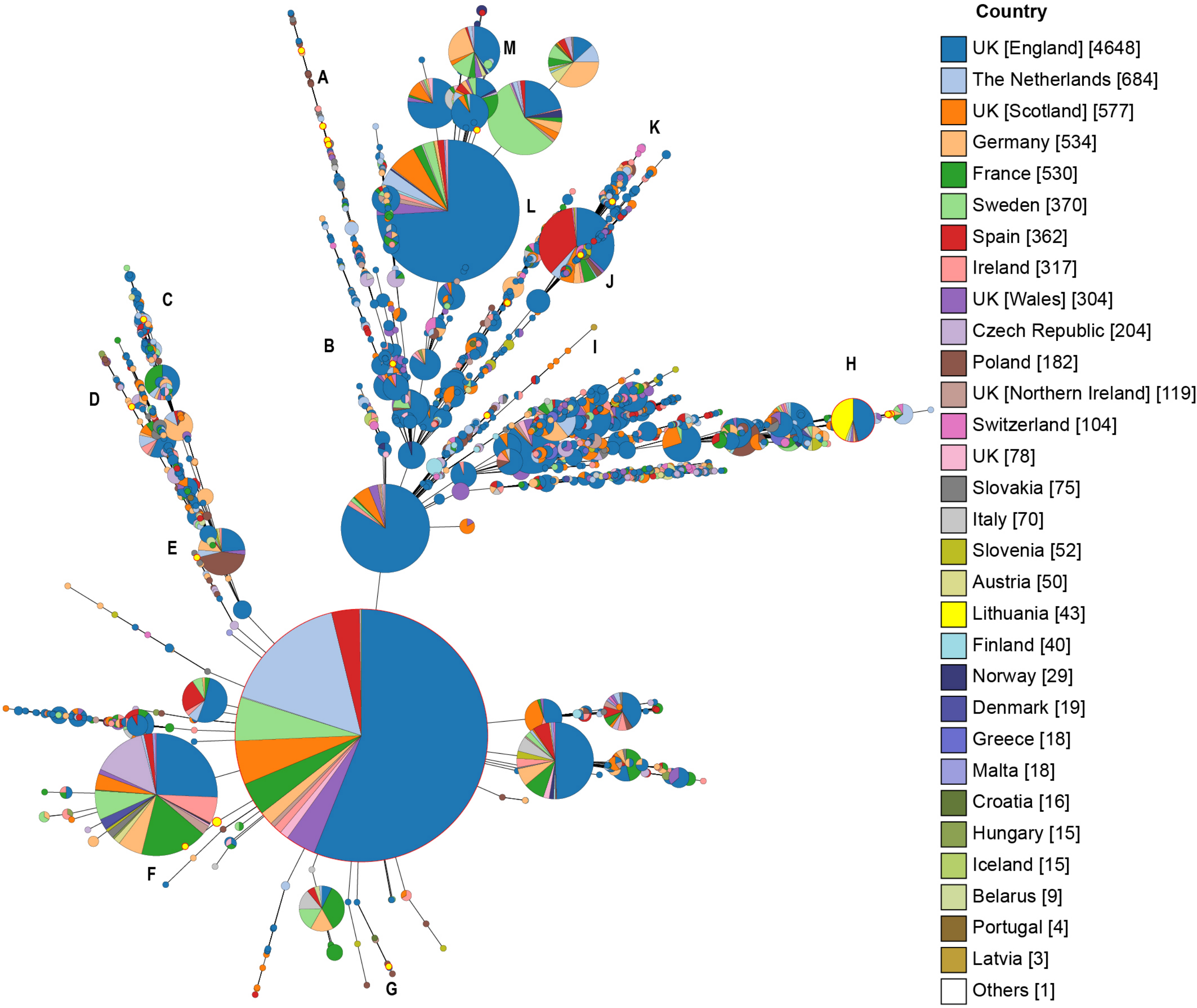

Supplementary Figure S1 The minimum spanning tree based on the results of cgMLST for publicly available *N. meningitidis* isolates. The MST is displayed using log-scale collapsed branches for allele distances of <100. The size of each circle is proportional to the number of strains it contains. The colors indicate the corresponding countries of isolation. The nodes with a red circle indicate those which include Lithuanian isolates. Each branch containing Lithuanian isolates is labeled **A** - **M** in the figure, with expanded branches shown in Supplementary Figure S2 to illustrate all branches (allele distances of ≥1) between Lithuanian isolates and the nearest root node with close relatives.

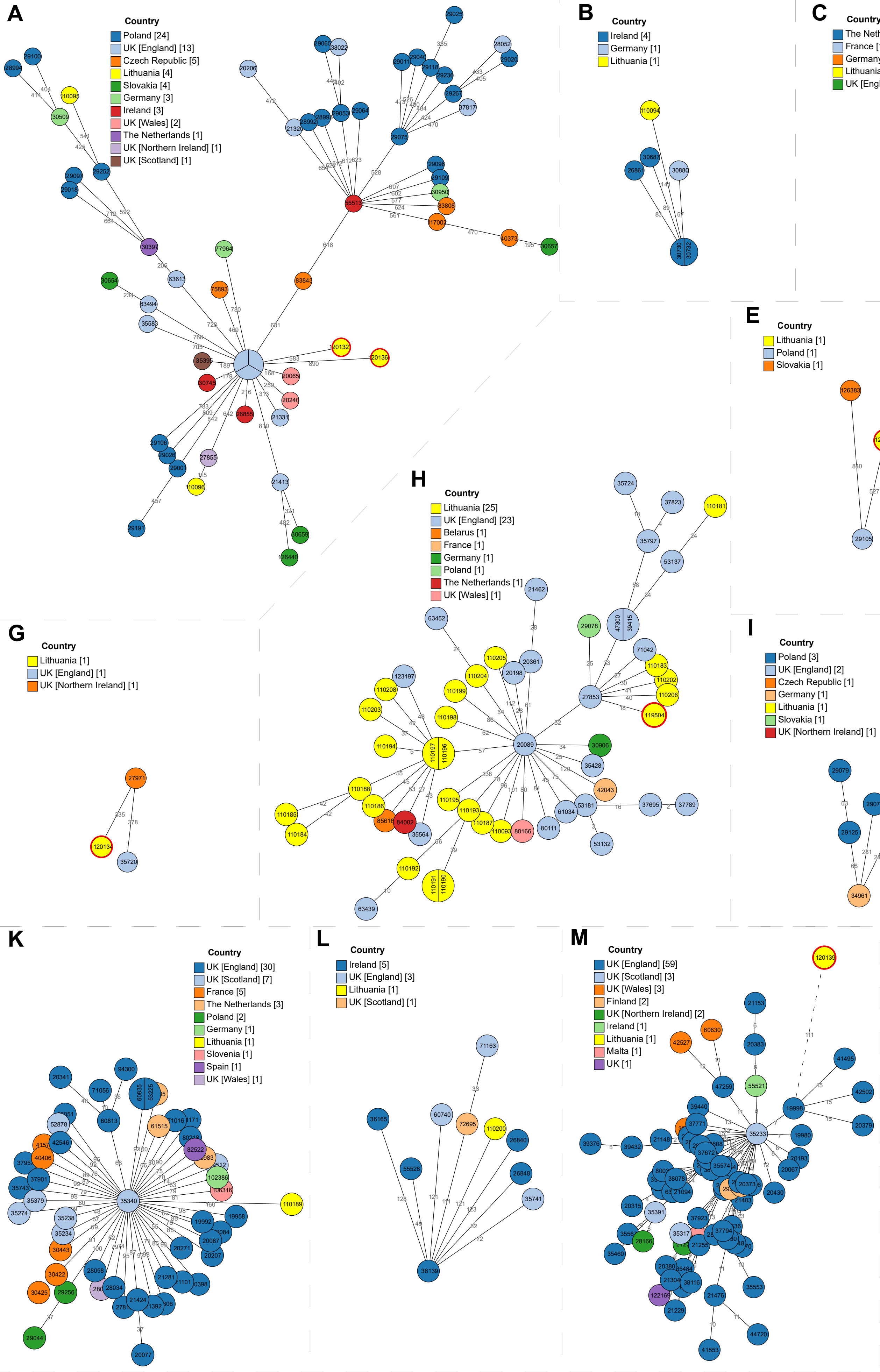

Supplementary Figure S2 The minimum spanning tree, generated from cgMLST data, illustrates the genetic relationships among Lithuanian isolates and closely related strains from the dataset depicted in Supplementary Figure S1. Each circle represents genetically related isolates, with circle size scaled to reflect strain count. The length of branches between each node represents the number of different alleles (out of cgMLST genes) that differ between two linked nodes. Node colors denote respective countries of isolation. Yellow circles bordered in red indicate isolates specifically analyzed in this study.
