# Supplementary material for: Genetic characterization of Neisseria meningitidis isolates recovered from patients with invasive meningococcal disease in Lithuania
Source: Front Cell Infect Microbiol. 2024 Oct 14;14:1432197. doi: 10.3389/fcimb.2024.1432197 (PMC11513629; doi:10.3389/fcimb.2024.1432197)
Supplement: Supplementary file 2 [file DataSheet2.pdf]

A

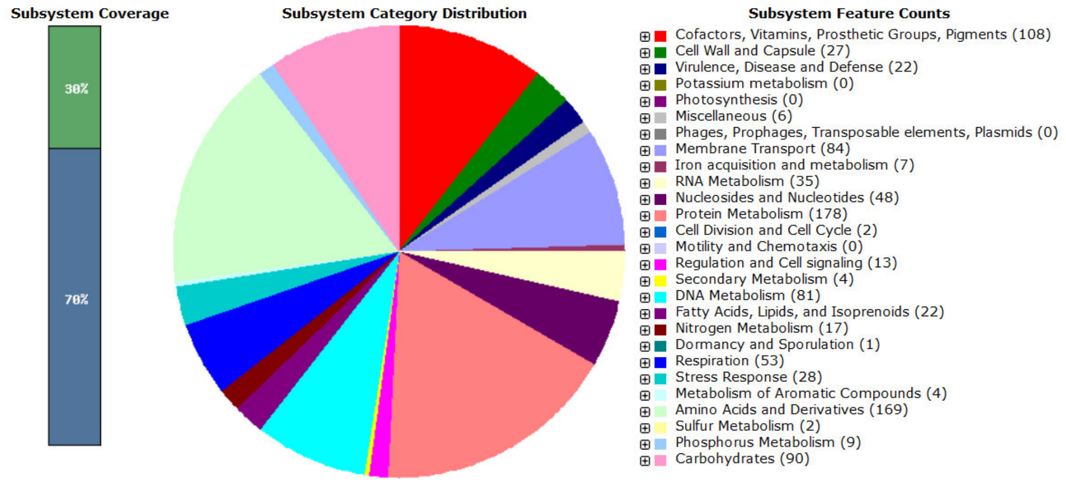
Subsystem Coverage
30%
70%
Subsystem Category Distribution
Subsystem Feature Counts
Cofactors, Vitamins, Prosthetic Groups, Pigments (108)
Cell Wall and Capsule (27)
Virulence, Disease and Defense (22)
Potassium metabolism (0)
Photosynthesis (0)
Miscellaneous (6)
Phages, Prophages, Transposable elements, Plasmids (0)
Membrane Transport (84)
Iron acquisition and metabolism (7)
RNA Metabolism (35)
Nucleosides and Nucleotides (48)
Protein Metabolism (178)
Cell Division and Cell Cycle (2)
Motility and Chemotaxis (0)
Regulation and Cell signaling (13)
Secondary Metabolism (4)
DNA Metabolism (81)
Fatty Acids, Lipids, and Isoprenoids (22)
Nitrogen Metabolism (17)
Dormancy and Sporulation (1)
Respiration (53)
Stress Response (28)
Metabolism of Aromatic Compounds (4)
Amino Acids and Derivatives (169)
Sulfur Metabolism (2)
Phosphorus Metabolism (9)
Carbohydrates (90)

B

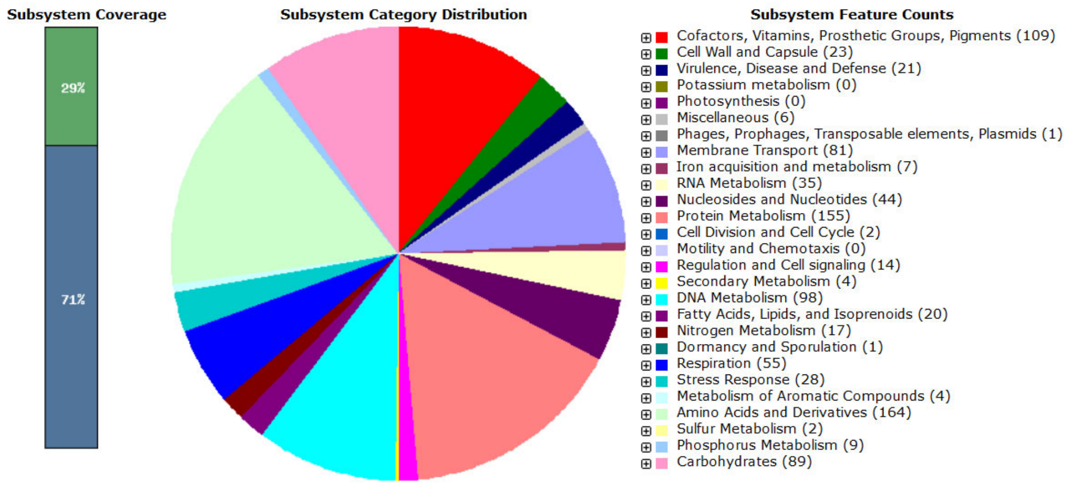
Subsystem Coverage
29%
71%
Subsystem Category Distribution
Subsystem Feature Counts
Cofactors, Vitamins, Prosthetic Groups, Pigments (109)
Cell Wall and Capsule (23)
Virulence, Disease and Defense (21)
Potassium metabolism (0)
Photosynthesis (0)
Miscellaneous (6)
Phages, Prophages, Transposable elements, Plasmids (1)
Membrane Transport (81)
Iron acquisition and metabolism (7)
RNA Metabolism (35)
Nucleosides and Nucleotides (44)
Protein Metabolism (155)
Cell Division and Cell Cycle (2)
Motility and Chemotaxis (0)
Regulation and Cell signaling (14)
Secondary Metabolism (4)
DNA Metabolism (98)
Fatty Acids, Lipids, and Isoprenoids (20)
Nitrogen Metabolism (17)
Dormancy and Sporulation (1)
Respiration (55)
Stress Response (28)
Metabolism of Aromatic Compounds (4)
Amino Acids and Derivatives (164)
Sulfur Metabolism (2)
Phosphorus Metabolism (9)
Carbohydrates (89)

C

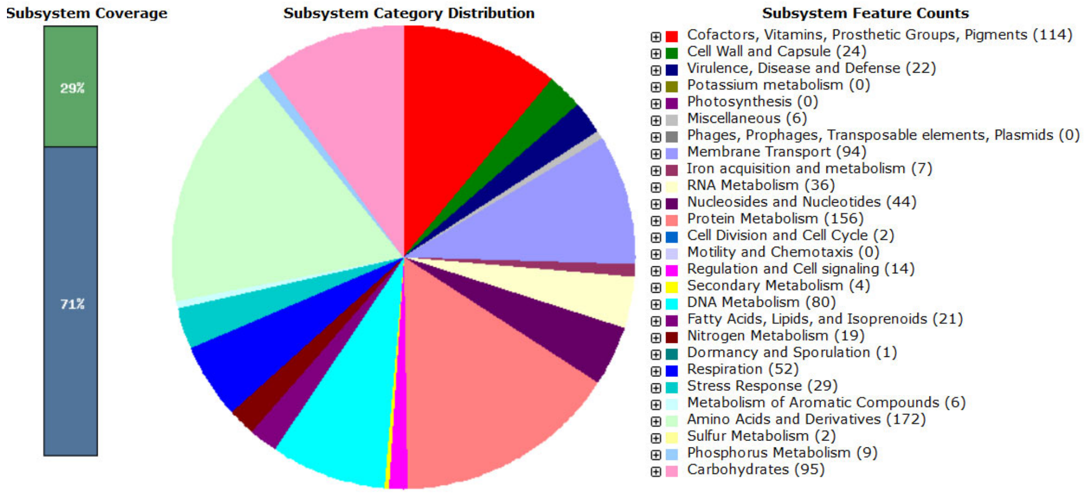
Subsystem Coverage
29%
71%
Subsystem Category Distribution
Subsystem Feature Counts
Cofactors, Vitamins, Prosthetic Groups, Pigments (114)
Cell Wall and Capsule (24)
Virulence, Disease and Defense (22)
Potassium metabolism (0)
Photosynthesis (0)
Miscellaneous (6)
Phages, Prophages, Transposable elements, Plasmids (0)
Membrane Transport (94)
Iron acquisition and metabolism (7)
RNA Metabolism (36)
Nucleosides and Nucleotides (44)
Protein Metabolism (156)
Cell Division and Cell Cycle (2)
Motility and Chemotaxis (0)
Regulation and Cell signaling (14)
Secondary Metabolism (4)
DNA Metabolism (80)
Fatty Acids, Lipids, and Isoprenoids (21)
Nitrogen Metabolism (19)
Dormancy and Sporulation (1)
Respiration (52)
Stress Response (29)
Metabolism of Aromatic Compounds (6)
Amino Acids and Derivatives (172)
Sulfur Metabolism (2)
Phosphorus Metabolism (9)
Carbohydrates (95)

D

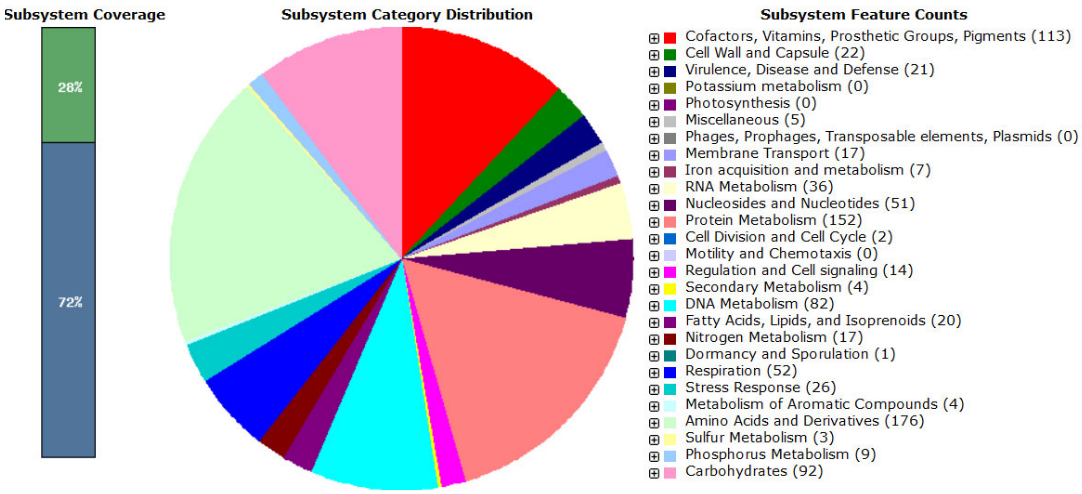
Subsystem Coverage
28%
72%
Subsystem Category Distribution
Subsystem Feature Counts
Cofactors, Vitamins, Prosthetic Groups, Pigments (113)
Cell Wall and Capsule (22)
Virulence, Disease and Defense (21)
Potassium metabolism (0)
Photosynthesis (0)
Miscellaneous (5)
Phages, Prophages, Transposable elements, Plasmids (0)
Membrane Transport (17)
Iron acquisition and metabolism (7)
RNA Metabolism (36)
Nucleosides and Nucleotides (51)
Protein Metabolism (152)
Cell Division and Cell Cycle (2)
Motility and Chemotaxis (0)
Regulation and Cell signaling (14)
Secondary Metabolism (4)
DNA Metabolism (82)
Fatty Acids, Lipids, and Isoprenoids (20)
Nitrogen Metabolism (17)
Dormancy and Sporulation (1)
Respiration (52)
Stress Response (26)
Metabolism of Aromatic Compounds (4)
Amino Acids and Derivatives (176)
Sulfur Metabolism (3)
Phosphorus Metabolism (9)
Carbohydrates (92)

E

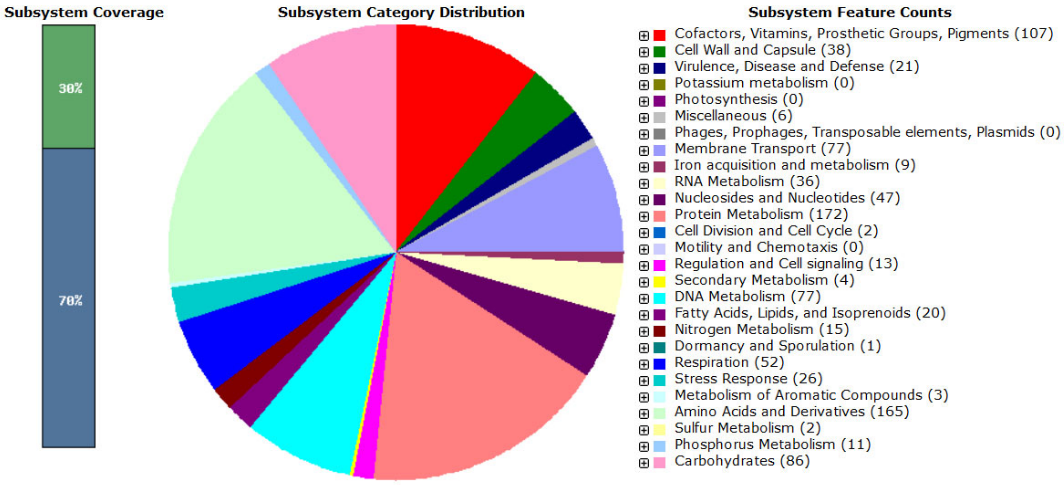
Subsystem Coverage
30%
70%
Subsystem Category Distribution
Subsystem Feature Counts
Cofactors, Vitamins, Prosthetic Groups, Pigments (107)
Cell Wall and Capsule (38)
Virulence, Disease and Defense (21)
Potassium metabolism (0)
Photosynthesis (0)
Miscellaneous (6)
Phages, Prophages, Transposable elements, Plasmids (0)
Membrane Transport (77)
Iron acquisition and metabolism (9)
RNA Metabolism (36)
Nucleosides and Nucleotides (47)
Protein Metabolism (172)
Cell Division and Cell Cycle (2)
Motility and Chemotaxis (0)
Regulation and Cell signaling (13)
Secondary Metabolism (4)
DNA Metabolism (77)
Fatty Acids, Lipids, and Isoprenoids (20)
Nitrogen Metabolism (15)
Dormancy and Sporulation (1)
Respiration (52)
Stress Response (26)
Metabolism of Aromatic Compounds (3)
Amino Acids and Derivatives (165)
Sulfur Metabolism (2)
Phosphorus Metabolism (11)
Carbohydrates (86)

F

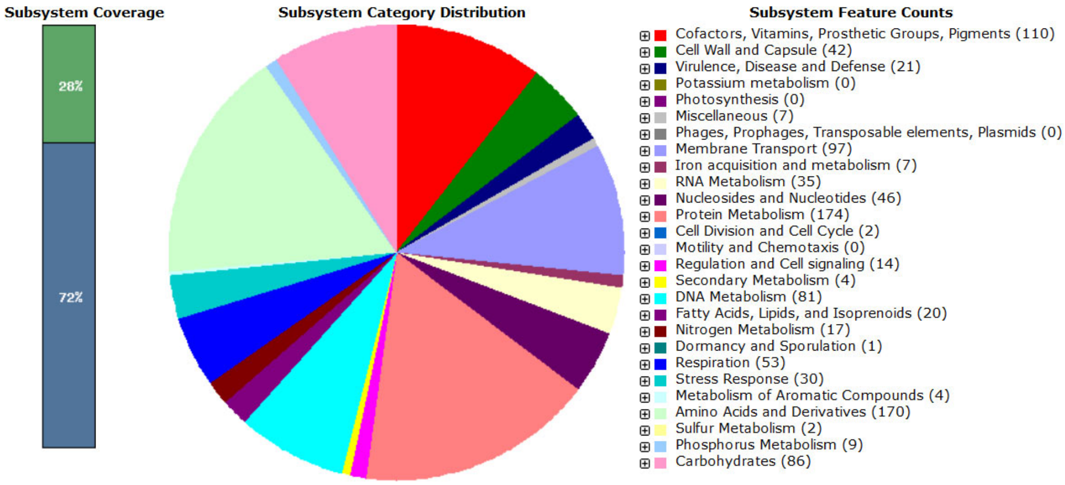
Subsystem Coverage
28%
72%
Subsystem Category Distribution
Subsystem Feature Counts
Cofactors, Vitamins, Prosthetic Groups, Pigments (110)
Cell Wall and Capsule (42)
Virulence, Disease and Defense (21)
Potassium metabolism (0)
Photosynthesis (0)
Miscellaneous (7)
Phages, Prophages, Transposable elements, Plasmids (0)
Membrane Transport (97)
Iron acquisition and metabolism (7)
RNA Metabolism (35)
Nucleosides and Nucleotides (46)
Protein Metabolism (174)
Cell Division and Cell Cycle (2)
Motility and Chemotaxis (0)
Regulation and Cell signaling (14)
Secondary Metabolism (4)
DNA Metabolism (81)
Fatty Acids, Lipids, and Isoprenoids (20)
Nitrogen Metabolism (17)
Dormancy and Sporulation (1)
Respiration (53)
Stress Response (30)
Metabolism of Aromatic Compounds (4)
Amino Acids and Derivatives (170)
Sulfur Metabolism (2)
Phosphorus Metabolism (9)
Carbohydrates (86)

G

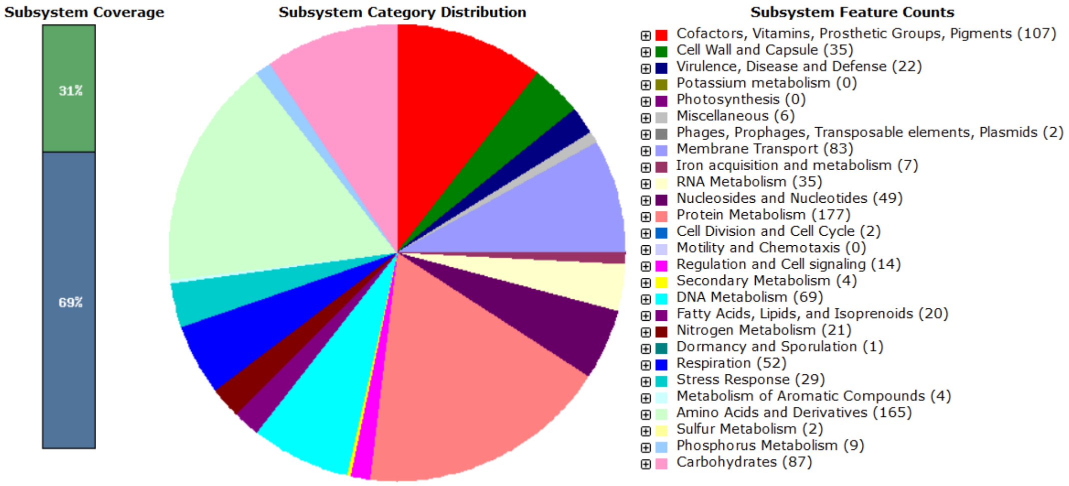

Subsystem Coverage
31%
69%
Subsystem Category Distribution
Subsystem Feature Counts
Cofactors, Vitamins, Prosthetic Groups, Pigments (107)
Cell Wall and Capsule (35)
Virulence, Disease and Defense (22)
Potassium metabolism (0)
Photosynthesis (0)
Miscellaneous (6)
Phages, Prophages, Transposable elements, Plasmids (2)
Membrane Transport (83)
Iron acquisition and metabolism (7)
RNA Metabolism (35)
Nucleosides and Nucleotides (49)
Protein Metabolism (177)
Cell Division and Cell Cycle (2)
Motility and Chemotaxis (0)
Regulation and Cell signaling (14)
Secondary Metabolism (4)
DNA Metabolism (69)
Fatty Acids, Lipids, and Isoprenoids (20)
Nitrogen Metabolism (21)
Dormancy and Sporulation (1)
Respiration (52)
Stress Response (29)
Metabolism of Aromatic Compounds (4)
Amino Acids and Derivatives (165)
Sulfur Metabolism (2)
Phosphorus Metabolism (9)
Carbohydrates (87)

H

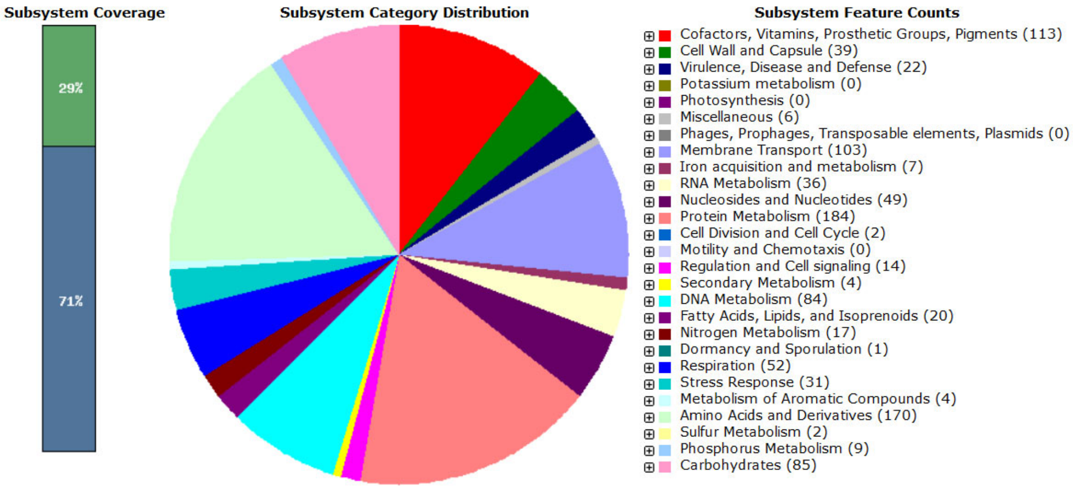

Subsystem Coverage
29%
71%
Subsystem Category Distribution
Subsystem Feature Counts
Cofactors, Vitamins, Prosthetic Groups, Pigments (113)
Cell Wall and Capsule (39)
Virulence, Disease and Defense (22)
Potassium metabolism (0)
Photosynthesis (0)
Miscellaneous (6)
Phages, Prophages, Transposable elements, Plasmids (0)
Membrane Transport (103)
Iron acquisition and metabolism (7)
RNA Metabolism (36)
Nucleosides and Nucleotides (49)
Protein Metabolism (184)
Cell Division and Cell Cycle (2)
Motility and Chemotaxis (0)
Regulation and Cell signaling (14)
Secondary Metabolism (4)
DNA Metabolism (84)
Fatty Acids, Lipids, and Isoprenoids (20)
Nitrogen Metabolism (17)
Dormancy and Sporulation (1)
Respiration (52)
Stress Response (31)
Metabolism of Aromatic Compounds (4)
Amino Acids and Derivatives (170)
Sulfur Metabolism (2)
Phosphorus Metabolism (9)
Carbohydrates (85)

I

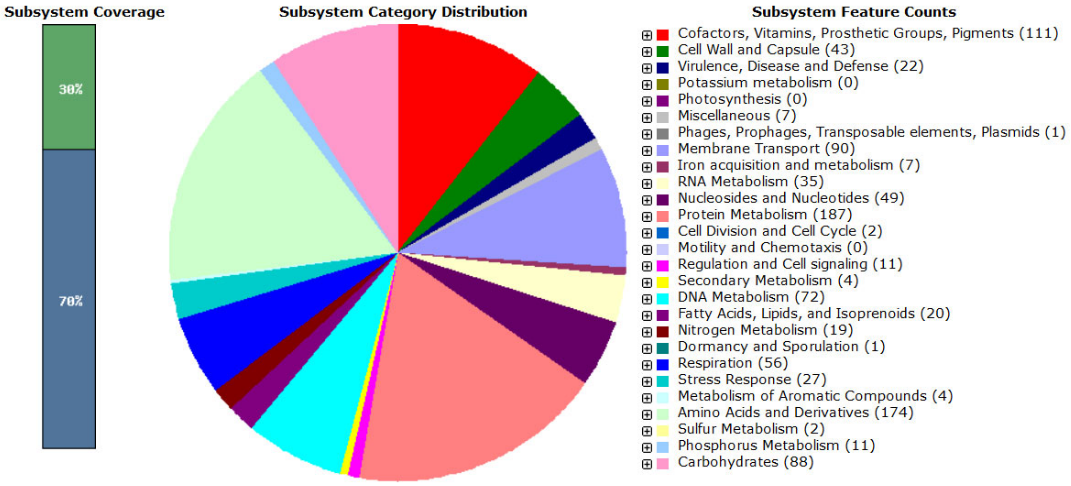

Subsystem Coverage
30%
70%
Subsystem Category Distribution
Subsystem Feature Counts
Cofactors, Vitamins, Prosthetic Groups, Pigments (111)
Cell Wall and Capsule (43)
Virulence, Disease and Defense (22)
Potassium metabolism (0)
Photosynthesis (0)
Miscellaneous (7)
Phages, Prophages, Transposable elements, Plasmids (1)
Membrane Transport (90)
Iron acquisition and metabolism (7)
RNA Metabolism (35)
Nucleosides and Nucleotides (49)
Protein Metabolism (187)
Cell Division and Cell Cycle (2)
Motility and Chemotaxis (0)
Regulation and Cell signaling (11)
Secondary Metabolism (4)
DNA Metabolism (72)
Fatty Acids, Lipids, and Isoprenoids (20)
Nitrogen Metabolism (19)
Dormancy and Sporulation (1)
Respiration (56)
Stress Response (27)
Metabolism of Aromatic Compounds (4)
Amino Acids and Derivatives (174)
Sulfur Metabolism (2)
Phosphorus Metabolism (11)
Carbohydrates (88)

J

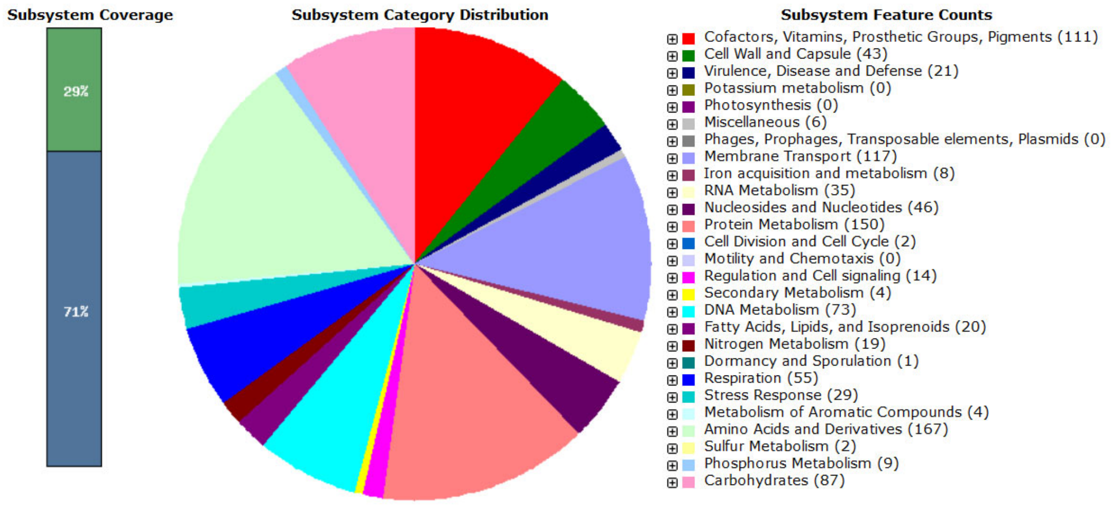

Supplementary Figure S3. Seed viewer obtained from RAST (Rapid Annotation using Subsystem Technology) (Overbeek et al., 2014) analysis of the whole genome sequence of *N. meningitidis* strains. (**A**) Isolate ID 119504; (**B**) Isolate ID 120132; (**C**) Isolate ID 120133; (**D**) Isolate ID 120134; (**E**) Isolate ID 120135; (**F**) Isolate ID 120136; (G) Isolate ID 120137; (H) Isolate ID 120138; (**I**) Isolate ID 120139; (**J**) Isolate ID 120140.
