# Supplementary figures and images for: Genetic characterization of Neisseria meningitidis isolates recovered from patients with invasive meningococcal disease in Lithuania
Source: Front Cell Infect Microbiol. 2024 Oct 14;14:1432197. doi: 10.3389/fcimb.2024.1432197 (PMC11513629; doi:10.3389/fcimb.2024.1432197)

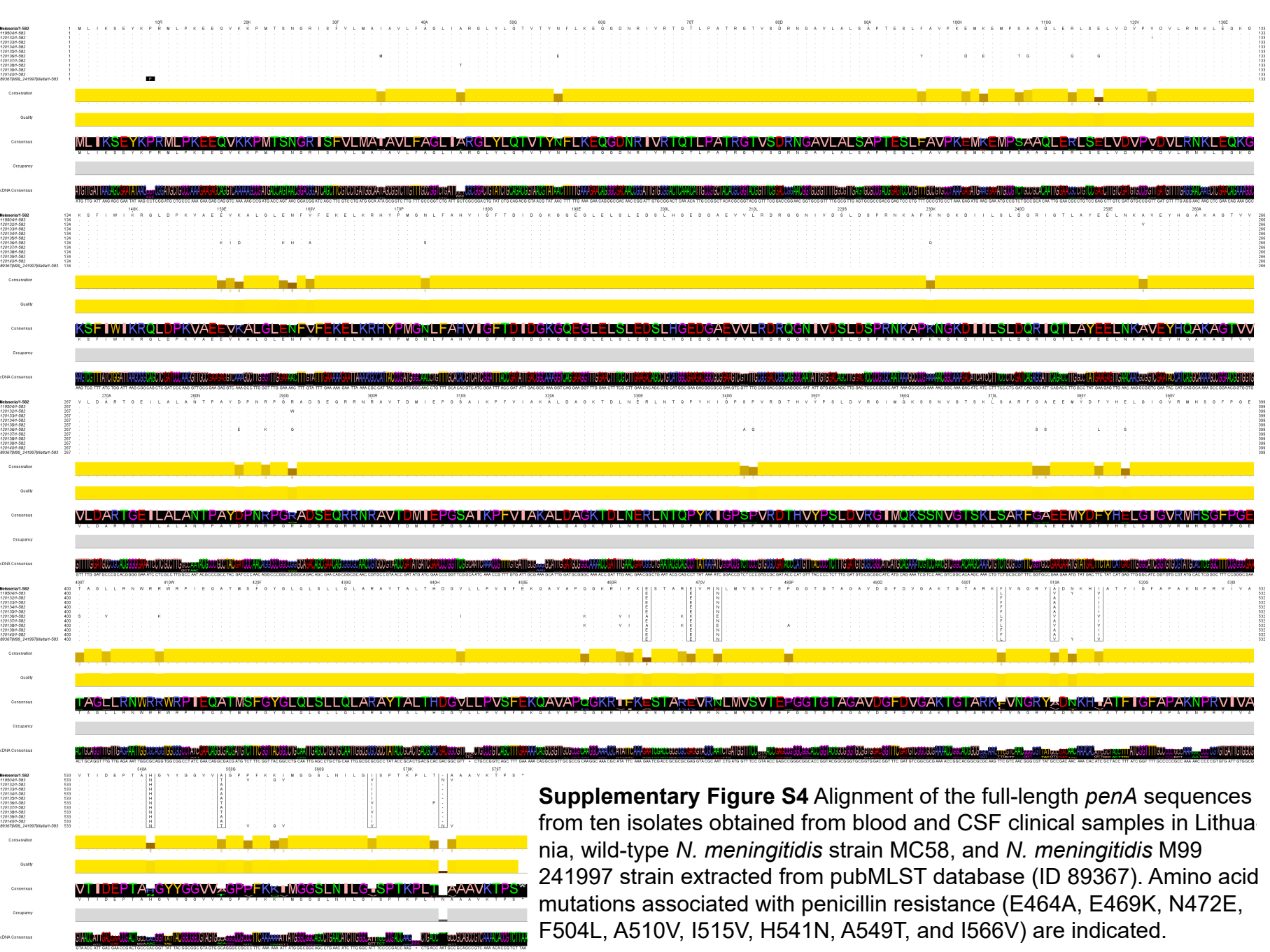

Supplement: Supplementary file 3 [file DataSheet3.pdf]
